# Patient and public involvement in the co-design and assessment of unobtrusive sensing technologies for care at home: a user-centric design approach

**DOI:** 10.1186/s12877-024-05674-y

**Published:** 2025-01-21

**Authors:** Jenny Sharma, Nazia Gillani, Imran Saied, Aaesha Alzaabi, Tughrul Arslan

**Affiliations:** 1 Advanced Care Research Centre (ACRC), University of Edinburgh, Edinburgh, UK; 2 School of Engineering, The University of Edinburgh, Edinburgh, EH9 3FF UK

**Keywords:** Patient and public involvement, User-centred design, Home-based care, Unobtrusive assistive technology, Aging in place, Wi-Fi and radar sensors, Remote monitoring

## Abstract

**Background:**

There is growing interest in developing sensing solutions for remote health monitoring to support the safety and independence of older adults. To ensure these technologies are practical and relevant, people-centred design is essential. This study aims to explore the involvement of various stakeholders across different developmental stages to inform the design and assess the capabilities of unobtrusive sensing solutions being developed as part of the Advanced Care Research Centre (ACRC), Edinburgh, UK.

**Methods:**

This study was conducted in two phases. In Phase I (Ideation), discussions were held with stakeholders (*n* = 19), including senior geriatricians (*n* = 2), healthcare and care home professionals (*n* = 4), PPI experts (*n* = 2), researchers (*n* = 4) and public members aged 65 and above from the ACRC Patient and Public Involvement (PPI) Network (*n* = 7). The goal was to identify clinically significant health parameters and design preferences. Based on this, prototypes of unobtrusive sensors for monitoring movement, hydration, and respiration were developed. In Phase II (Development and Co-Design), an in-person PPI workshop was conducted with PPI experts (*n* = 2), researchers (*n* = 4) and PPI members (*n* = 8). The developed prototypes were demonstrated, and qualitative feedback was collected through focus group discussions on themes such as acceptability, usability, privacy, data sharing, and functionality enhancement.

**Results:**

Stakeholder input from Phase I emphasized the importance of non-contact sensing technologies that maintain privacy. Movement, hydration, and respiration were identified as critical health parameters. In Phase II, PPI members were optimistic about the prototypes, valuing their unobtrusive design and privacy-preserving features. Key themes identified included (1) the need for user-customized alarms, (2) clear data-sharing protocols, and (3) the importance of embedding sensors into familiar household objects. Suggestions for refining the prototypes included adding functionality for detecting deviations in daily routines and integrating feedback mechanisms for caregivers.

**Conclusions:**

Involving diverse stakeholders from the early stages of technology development enhanced the relevance and acceptability of unobtrusive sensing solutions. This study highlights the importance of integrating public perspectives into the design process. For successful implementation, developers of healthcare technologies should prioritize privacy, usability, and clear communication with end-users and caregivers.

**Supplementary Information:**

The online version contains supplementary material available at 10.1186/s12877-024-05674-y.

## Introduction

The world is seeing a demographic shift in the ageing population. According to the World Health Organization (WHO), the proportion of the global population aged 60 and above has increased from 12 to 22% from 2015 to 2020. It is estimated that in 2030, 1 in every 6 individuals will be 60 years and above [1]. This exponential growth is liable to cause a strain on the already burdened healthcare system. To support this demographic change, the care model needs to move from reactive to proactive through remote health monitoring, focusing on early interventions and prevention [2].

Ambient sensors and assistive technology are playing a key role in transforming the healthcare landscape enabling remote monitoring of daily activities and generating alarms when a deviation from the routine is sensed [3–5]. In addition to the wearable and ambient smart home and camera sensors, electromagnetic and radio frequency-based sensors are emerging as a reliable technology for remote health monitoring [6–10]. Moreover, these Wi-Fi and radar-based sensors are privacy-preserving, and their unobtrusive and non-contact nature does not restrict the movement or daily activities of the individual being monitored. However, while technological advancements are significant, ensuring their quality, relevance, and long-term impact, integrating patient and public involvement (PPI) at various stages of the design and development process. According to the National Institute for Health and Care Research (NIHR), PPI is defined as “doing research ‘with’ or ‘by’ people who use services rather than ‘to’, ‘about’ or ‘for’ them” [11, 12]. Notably, the literature mentions the benefits of collaborative design, which include seamless design, implementation, acceptability, and successful engagement after deployment for the intended users [13].

Though PPI is regarded as fundamental in developing and innovating digital healthcare technologies, the literature still needs studies that formally report PPI events and their involvement at different stages of technology development for caring for older adults and enhancing their independence in their homes [14]. A scoping review by Cluley et al. [15], which examined 87 studies, revealed that PPI is most common during the early stages of healthcare innovation, such as problem identification and invention. However, PPI involvement in later stages—such as adoption, diffusion, and long-term evaluation—remains underreported. Moreover, most studies (71%) focused on process and service innovations, with only less than 8% addressing technological advancements in healthcare. Specific focus areas include mental health (21%) and cancer care (9%), leaving other domains, such as care for older adults at home, less explored.

Among studies that involve PPI in digital healthcare technology, a notable emphasis has been on dementia care [16–18]. In contrast, others focus on the feasibility of hosting hybrid PPI events [19], evaluating health services during hospital discharge [20], or assessing frailty-monitoring technologies for older adults [21]. Recent research also underscores the importance of PPI in adopting computer-assisted diagnostic technologies, particularly its potential as an authoritative voice in regulatory review and healthcare adoption processes [22]. Collectively, these studies highlight the utility of PPI in ensuring the relevance of technological solutions.

Despite these advancements, significant gaps persist. Current literature often focuses on soliciting patient or public perspectives to inform decisions (consultation) [23] but less frequently reports on direct participation in decision-making processes at different stages of technology development and design process. Furthermore, very few studies comprehensively document the involvement of older adults and other key stakeholders—such as geriatricians, care home experts and other healthcare professionals—in healthcare technologies’ design and implementation. This lack of inclusion may contribute to the rejection or abandonment of technologies, often due to dissatisfaction, incompatibility, or a perceived lack of relevance to the needs [21].

Unlike much of the existing literature, which focuses primarily on early-stage consultations or limited end-user feedback, this study makes contributions to healthcare technology development by advancing the integration of various stakeholders, including patient and public members, in the ideation, co-design and assessment of these technologies for the independence and care of older adults. This user-centric approach enhances these technologies’ relevance, acceptability, and long-term feasibility.

Specifically, this study bridges critical gaps by systematically involving patients and public members in consultation to inform decisions and directly influence decision-making processes. This dual involvement addresses the underrepresentation of older adults and key stakeholders in the later stages of healthcare technology development, such as adoption, diffusion, and iterative improvement. The findings emphasize the critical importance of unobtrusive, privacy-preserving technologies in maintaining the dignity and independence of older adults. We developed Wi-Fi and radar-based sensors that align with real-world needs by focusing on parameters such as movement, hydration, and respiration, identified as clinically significant and user-prioritized. Notably, the in-person PPI workshop demonstrated how direct engagement in co-design enhances understanding and fosters acceptance of these technologies among end-users. Key contributions of this study include:

1. **Stakeholder Integration Across Stages**: A structured approach to involving older adults, geriatricians, and healthcare professionals in identifying health parameters, guiding design decisions, and evaluating prototypes.
2. **Advancing Co-Design**: Demonstrating the value of physical prototype demonstrations and iterative feedback sessions to refine sensing technologies, particularly in tailoring alarm systems, data-sharing protocols, and device usability.
3. **PPI Impact Evaluation**: Evaluating the impact of end-user participation and expert stakeholder consultation on the design, implementation, and successful adoption of sensing technologies in healthcare, with a focus on ensuring these innovations are tailored to meet patient needs, align with their values, and enhance user-centric healthcare solutions.

This work underscores the importance of meaningful stakeholder engagement in technology development and is a scalable model for advancing PPI practices in healthcare innovation. By addressing challenges such as abandonment due to incompatibility or lack of relevance, our study provides a pathway for designing user-centric technologies that are more likely to achieve widespread acceptance and sustained use. Furthermore, the findings contribute to a growing body of evidence supporting PPI’s role in bridging the gap between innovation and practical healthcare solutions, particularly for the ageing population.

By addressing these objectives, this study seeks to advance the role of PPI beyond consultation to include active participation, particularly in later stages of technology development, such as adoption and diffusion. In doing so, it aims to bridge the gap between technological innovation and practical implementation, ensuring that solutions are compatible with the needs and expectations of older adults and other stakeholders. This underlines the need for stronger PPI in later stages, such as adoption and diffusion, to ensure broader implementation and impact.

The sensing solutions are being developed as part of the Advanced Care Research Centre (ACRC), a multi-disciplinary research programme integrating the fields of social science, medicine, healthcare, engineering, informatics, and data science [24]. The ACRC seeks to develop and deliver data-driven, high-quality, and personalised care solutions to support independence and remote care for the ageing population. The current work focuses The project’s engineering team is developing the sensing solutions as part of ‘Integrated Technologies of Care’, a subpackage of the ACRC [25]. These include radio frequency and radar-based sensors that are being designed and tailored for an unobtrusive sensing platform which can monitor various health parameters, specifically movement parameters, respiration, and hydration, while individuals perform activities of daily living.

## Methods

### Design

To develop functional and practical technology for the care of older adults, it was imperative to comprehensively understand the needs, requirements, and concerns of both end-users and professional healthcare workers from the project’s inception. Therefore, in Phase I, the researchers (*n* = 4) and PPI experts (*n* = 2) actively engaged with the healthcare professionals (*n* = 6) and the public and patient members (*n* = 7). Through extensive discussions with healthcare professionals and PPI members, clinically significant health parameters were identified and prioritized for monitoring. The outcome of this initial phase provided a clear focus on quantitatively measuring individuals’ hydration, movement, and respiration. Additionally, the PPI members emphasised the importance of non-contact sensing solutions to preserve the privacy and dignity of individuals. This collaborative engagement laid the groundwork for the study’s development.

Phase II, conducted almost two years later, involved a formal in-person Public and Patient Involvement (PPI) event at the University of Edinburgh, UK, with 14 individuals participating. These included PPI experts (*n* = 2), researchers (*n* = 4) and the patient and public members (*n* = 8). In Phase II, the researchers demonstrated the technologies developed to the PPI members. Following the demonstration, group discussions were held for feedback and co-design for further improvements of the technologies, facilitated by the PPI experts.

### PPI network in the ACRC

Advanced Care Research Centre (ACRC), University of Edinburgh has an established PPI network [26]. The members include a diverse group of older adults who are experienced in providing input and feedback to researchers regarding health and social care research. The PPI members present expertise based on their lived experiences of ageing and care, along with their personal and professional skills from other aspects of their lives.

### PPI recruitment

For recruitment and involvement of the PPI members, invites through emails were sent with details of the event, its aims, and objectives. PPI members who had prior engagement with health and technology projects or expressed interest in improving care for older adults were invited. Those who accepted, according to their ease and availability, were invited to participate in the PPI events with details of the day’s venue, time, and activities. The details of the invites sent and the information shared with the public members about the venue and objectives of the PPI event are available as supplementary files (Appendices 2, 3 and 6). The criteria for people joining the ACRC Patient and Public Involvement Network consider themselves in later life, have experience in health and social care and are interested in using technology and data to improve healthcare. The Network is also open to family members or people who provide unpaid support to older adults to join and share their perspectives [26].

### Ideal focus group size

In the literature, 6 to 12 participants are considered as ideal size for focus groups [27, 28]. However, Quine et al. [29], suggest that for older adults the ideal focus group size is 5–6 participants. Groups larger than 6 members could impose challenges in eye contact and audibility. Similarly, a group size of less than four people could be less dynamic. Hence, in our study, all focus groups were of a maximum of six members including 2 researchers to take notes, record feedback and guide the discussions.

### Ethics statement and PPI reporting guidelines - (GRIPP2 long form Checklist)

In line with the explicit guidance from the National Institute for Health and Care Research (NIHR) [30] and the UK Health Research Authority (HRA) tool [31], the patient and public involvement activity requires no ethical approval or consent from the attendees. According to the NIHR, if researchers “collect opinions rather than study data, the activity is likely an involvement activity”, and for that, the researchers “do not need ethics to conduct an involvement activity” [30]. The NIHR states, “Patient and public involvement should inform research questions or research design with Public and Patient (and carer) opinions [30]”. Moreover, it also explicitly states that if “opinions are collected rather than study data, the activity is likely an involvement activity; for example, asking for feedback on a questionnaire counts as involvement as long as you do not ask for or record the public contributor’s responses to the questions but their opinions on the suitability/wording of the questions” [30]. As these were the primary objectives of the study, hence, in line with these guidelines, ethical approval and consent were not required. However, to validate the unobtrusive sensors developed by the engineering researchers, the studies were approved by the University of Edinburgh’s School of Engineering ethical review board, reference numbers 2109414 and 1908056.

The PPI event has been reported according to the guidelines specified by GRIPP2 (Guidance for Reporting Involvement of Patients and the Public) long form [32]. This is an international consensus for the agreed checklist while reporting a PPI study. The information presented in this paper to map the requirements and checklist for GRIPP2 long form is provided in the supplementary material (Appendix 1).

### PPI training sessions for researchers

Before the PPI events, the researchers underwent PPI training by the ACRC PPI coordinator and team. The training emphasised the importance and the need of involving public members to do the research ‘with the users’ rather than ‘for them’. Researchers were also briefed on how to communicate effectively and understand the use of appropriate technical depth in language while communicating with the PPI members. The questions that were chosen to guide the discussion sessions in focus group sessions were collectively formulated by the PPI expert team and the researchers. Moreover, an interactive session for effective note-taking and recording the responses of the PPI members while stating relevant quotes during focus group sessions was also part of the training.

### Phase I: ideation stage

**Fig. 1:**
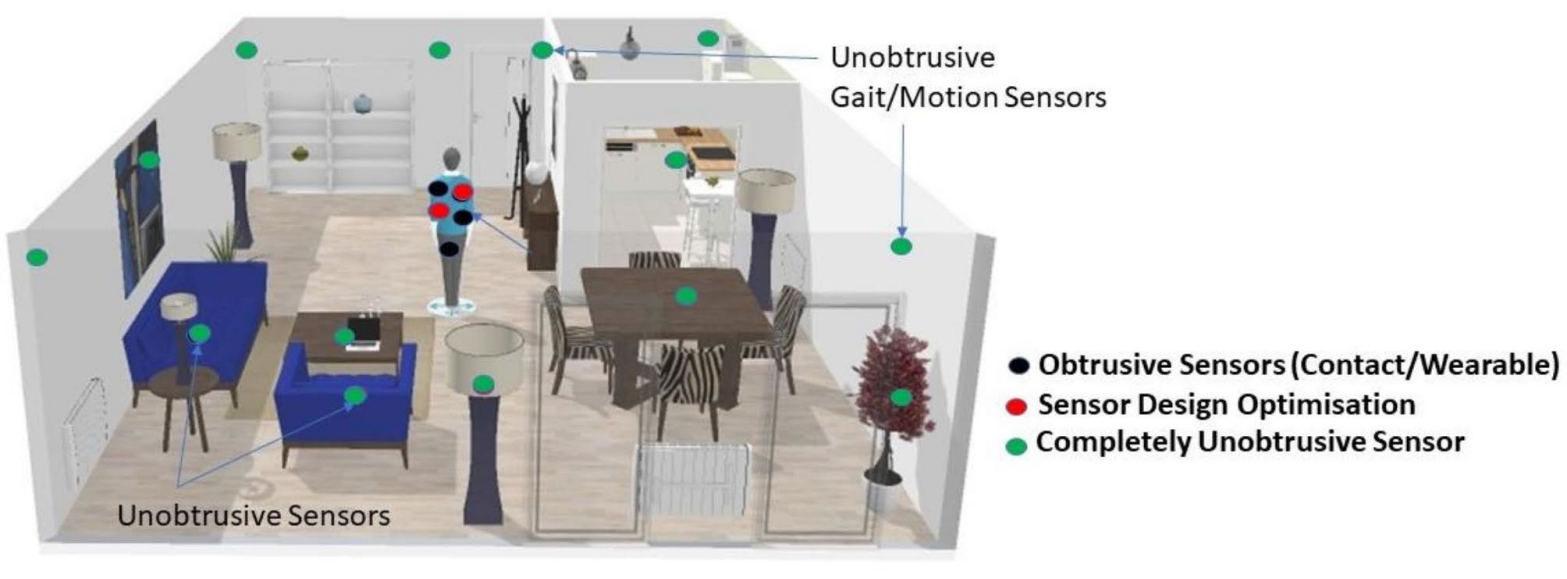
Initial idea for the unobtrusive sensing platform for remote health monitoring (Phase I)

At the beginning of the research project, the researchers had several discussions with healthcare professionals (*n* = 6) and PPI members (*n* = 7) along with researchers (*n* = 4) and PPI experts (*n* = 2) to determine the extent of sensing technologies used for care applications and the approach used in developing and deploying the technologies. For Phase I, participants were selected based on their expertise and involvement in geriatric care or related research. These included senior academic geriatricians with more than 20 years of experience (*n* = 2), the medical and technology lead at local care homes (*n* = 2), and experienced healthcare professionals from the Primary Palliative Care Research Group at The University of Edinburgh, UK (*n* = 2), one of whom is now retired. This discussion led to the initial stage of PPI work. Following these discussions, focus group meetings were held with the PPI members to discuss the identified health parameters and technology design choices. This was done to seek guidance on which parameters should be focused on first and what kind of technology older adults prefer. These meetings were held at the end of the year 2020 and the start of 2021; hence, they were all conducted online on Microsoft Teams due to Covid restrictions. During the meeting, researchers shared their findings on the various health parameters identified and their initial sensor design approach to capture them. Some sensor technologies focused on wearables, while others focused on unobtrusive sensors. Figure 1 shows the initial idea for the sensing platform to the stakeholders, including clinicians, healthcare professionals, and PPI members.

### Phase II: developmental stage and the in-person PPI event

The second phase of the PPI work took place two years later. It involved demonstrating the developed devices to more members of the public and potential users of the technology. An email invitation was sent to 17 ACRC Patient and Public Involvement Network members who lived in or near Edinburgh, of whom nine responded that they would like to attend the event. One member had to drop out on the day of the event, leaving us with five males and three females attending the workshop. A total of 14 people participated in the in-person workshop, including PPI experts (*n* = 2), researchers (*n* = 4), and patient and public members (*n* = 8).

The PPI event was organized to achieve the following objectives:

1. To demonstrate the sensing solution platform and the developed prototypes to the PPI members.
2. To assess the acceptability and feasibility of the sensing technologies.
3. To codesign the next stage of the sensor development, specifically focussing on:

- raising and communicating alarms in case of any deviation in the parameters from normality and.
- further tailoring the study design and technologies to make them even more effective and relevant for the intended users.

The in-person PPI event consisted of a demonstration of the sensing technologies and their capabilities. This was followed by two focus group discussion sessions. The sensing solutions demonstrated consisted of three main parameters being measured and quantified:

1. Unobtrusive Hydration Monitoring – sensors embedded in furniture (e.g., seat cushions, bed) to monitor changes in the body’s hydration.
2. Unobtrusive Movement monitoring – sensors embedded in household objects (e.g., vases, clocks, etc.) to monitor activities and quantify movements during eating walking etc.
3. Unobtrusive respiration monitoring -sensors placed on walls to monitor respiration rate.

### Demonstration

**Fig. 2:**
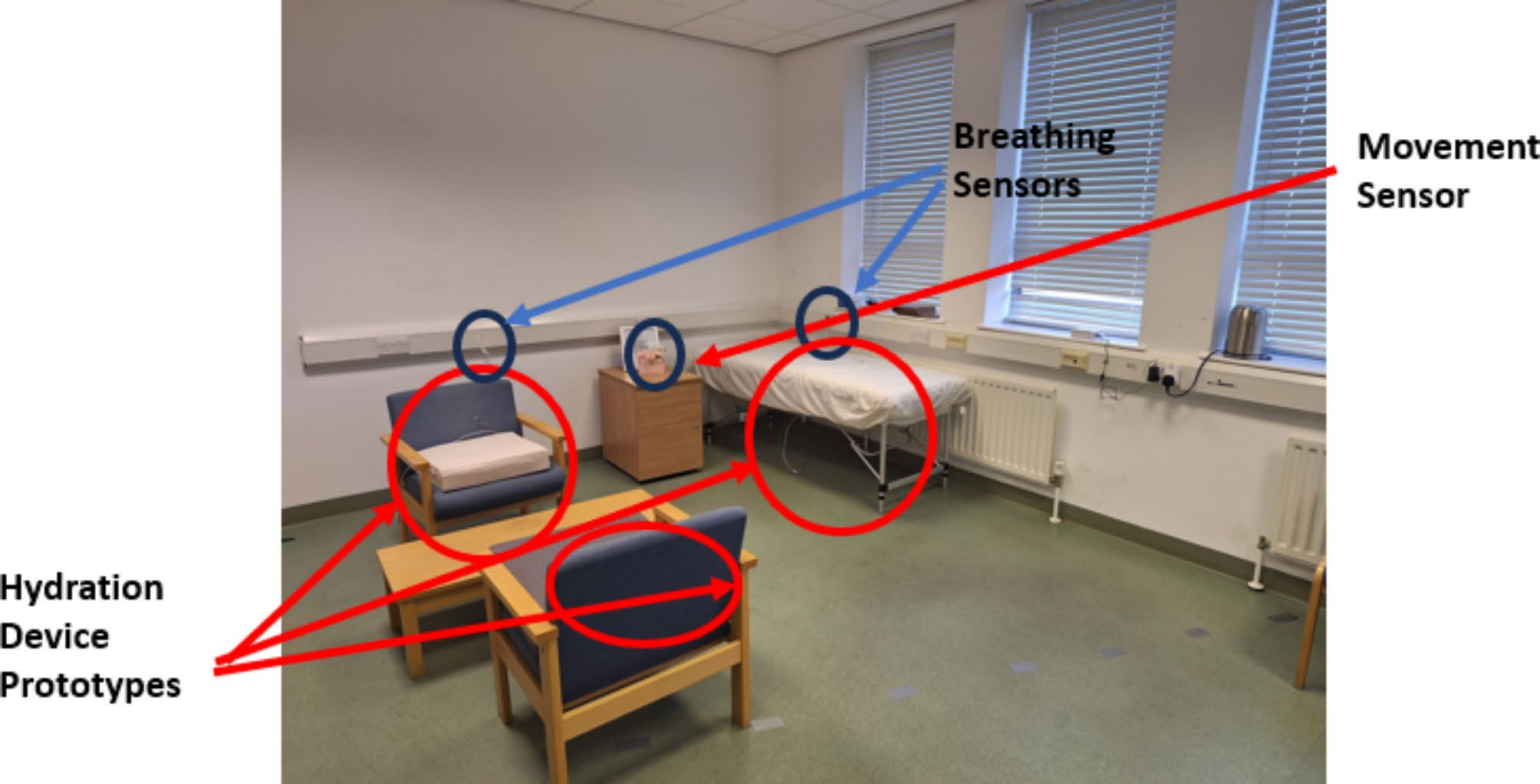
Practical demonstration of the developed unobtrusive sensing platform for remote health monitoring (Phase II)

The aim of the demonstration was twofold. The first was to familiarise the PPI members with the sensors, their visual aspects and how they can be integrated into a home environment for example by embedding them in home furniture. The second aim was to show the raw data captured and how signal processing can generate valuable insights into the health status of an individual. This was done to demonstrate that the data being captured will preserve an individual’s privacy by not capturing any distinct data that may be a reason for privacy invasion. The demonstration included a brief presentation on the three parameters being measured, their clinical significance and how these parameters can benefit both long- and short-term routine care monitoring for people at home. The presentation slides are available as supplementary material (Appendix 7). This was followed by a practical demonstration of the sensor set-up, how readings are taken, and the format of the data captured. Figure 2 illustrates the developed unobtrusive sensing platform and the available space where the experiments are performed to validate the sensors.

### Focus group discussions

After the demonstration, the PPI members were divided into two focus groups, each with six participants. A PPI expert and two researchers facilitated each group discussion, using preplanned goal-oriented questions to guide the conversation. The researchers documented the feedback by recording and transcribing the discussions for further analysis. Thematic analysis of the transcribed data was performed following the approach outlined by Nowell et al. [33]. This analysis adopted a deductive, a priori approach, applying a top-down methodology with predetermined codes [34]. The discussion questions were organized into two main themes to ensure a structured and focused exploration of the participants’ feedback. This approach enabled the researchers to systematically gather insights to refine the technology based on the input provided.

The first discussion session focussed on gathering feedback and suggestions on the developed sensing solutions and their capabilities. While the latter focussed on the co-design. The questions asked are detailed in Table 1.

**Table 1:** Questions for focus group discussion sessions

Type of Question				Question Statement as asked/ shown to PPI members
***Discussion Session – I***				
1	**Feedback**	*Positive*		What aspects of the sensors do you particularly like?
2		*Concerns*		What concerns do you have about the sensors?
3		*Suggestion*		How can we modify these sensors to make them more valuable to older adults and unpaid carers?
***Discussion Session – II***				
1	**Co-Design**		*Alarm/data sharing Preference*	What would you like the sensors to do if they record a change from normal hydration or movement patterns?
2			*Enhancing Study Design*	Are there any specific scenarios/ activities in everyday life during which these sensors would be particularly beneficial?

### Concluding the PPI event and collating findings

Following the discussion sessions, participants were given contact information to be involved in more PPI events. A feedback form was also distributed to all participants to gather their feedback for the PPI event. The form included questions designed to assess participants’ experiences, including their perceptions of the clarity of the pre-workshop information, opportunities to contribute during discussions, and the overall value of their contributions. Participants were also asked to evaluate how useful and informative they found the PPI event, the depth and detail of the discussions, and whether they felt the workshop met their expectations. Open-ended questions allowed participants to expand on their responses and provide suggestions for improvements or additional topics they would like addressed in future workshops. The feedback collected from these forms served two key purposes: first, to evaluate the event’s success in achieving its objectives, and second, to identify areas for improvement to enhance future PPI sessions. This systematic collection of feedback was essential for ensuring that the participants’ voices were heard during the discussions and used to refine the collaborative engagement process.

After the event, researchers compiled their notes, which were transcribed and transferred into a single shared Google document. In the subsequent analysis, the lead researcher thoroughly reviewed the notes and developed an initial coding template based on the recurring themes and key points discussed during the sessions. Over several meetings, the team of six researchers and two PPI experts collaboratively reviewed the notes and codes, identifying common themes and fundamental findings. The coding process was iterative, with the researchers continuously refining the codes as new patterns and insights emerged. The lead researcher carefully reviewed and condensed the list of codes, merging those that addressed similar concepts to ensure coherence and clarity.

Once the coding structure was finalized, the team grouped related concepts into sub-themes. This grouping process involved detailed discussions among the researchers and PPI experts to ensure consensus on the themes and to capture all primary concerns and essential co-design aspects highlighted by the participants. The lead researcher rigorously re-evaluated and refined the themes to ensure they accurately captured the breadth and depth of the discussions and findings from the focus groups. This iterative process aimed to uphold methodological rigour and ensure that the themes reflected the participants’ perspectives. Finally, illustrative quotes were meticulously selected to contextualize and substantiate each theme, providing authentic insights into participants’ views and aligning the findings with the co-design objectives of the PPI event.

## Results

### Phase I: ideation stage

#### Deciding parameters

Discussions with senior geriatrics and other healthcare professionals from the Primary Palliative Care Research Group led to identifying important health parameters from a care perspective. The details of the output from this phase and the conversations between the mentioned healthcare professionals are provided in the supplementary files as Appendix 8. These discussions highlighted essential parameters such as temperature, blood pressure, heart rate, hydration, continence, and blood oxygen saturation levels, typically measured via a fingertip pulse oximeter and other vital signs. Additionally, monitoring and predicting adverse events, particularly falls, were recognized as critical.

The identified health parameters were then shared with the PPI members. The PPI members provided feedback and guidance on (1) what primary health parameters to focus on and (2) what important sensor design considerations to follow. A PPI member said that

…. *We agree that the parameters identified are important. However*,* the need to capture respiration rate*,* our specific movement or activities*,* and hydration are significant…. for example*,* in the case of activities*,* monitoring eating and drinking routines or walking patterns*,* going to the toilet*,* and sleep disturbances etc. will be beneficial*….

In addition, they identified that these parameters are fundamental in monitoring people in later life, especially those with multimorbidity who require active management of various conditions. For example, in the case of movements, the primary concern was falls and the fear of falls. One of the PPI members stated,

“*The major concern we have is falling. We fear losing balance*,* especially while rising from the chair or bed*”.

Another member added,

*If I keep on sitting for long periods watching TV and then I remember that I must go to the toilet*,* the sudden standing up makes me dizzy*,* and it feels like I will fall while rushing to the bathroom*.

One PPI member asked,

*Will your sensors be capable of assessing or quantifying how my tremors vary throughout the day when I eat*,* grab a glass of water*,* or even use the remote control while watching the TV? Given that your sensors could quantify and continuously monitor my tremors throughout the day and week*,* it would be good to tell my GP on the next visit about the treatment’s effectiveness quantitively instead of just explaining what I observed about myself*.

Another consistent concern of many caregivers and PPI members was related to hydration. Most caregivers stated,

*When caring for their loved one*,* they are not sure if their family member drank enough water or perhaps*,* they drink too much*?

A carer at the care home and lead nurse in the falls unit of a local hospital in Edinburgh Uk stated:

*Dehydrated individuals are more likely to experience a fall/ Hence*,* its very important to monitor hydration levels so that we can take care of the person under observation in a better way and hopefully avoid accidents*.

Those who had respiration problems or were still experiencing the aftereffects of COVID-19 stated,

*The continuous monitoring of our respiration rate is of paramount importance as we all know we just are recovering from COVID-19. If people like us could have technologies that could tell us if our breathing is normal….that would be good!*

### Choice and preferences for technology and sensing systems

For technologies, the PPI members stated that they preferred unobtrusive sensors over wearables. This is because non-contact unobtrusive sensors will enable older adults to carry out daily activities without hindrance. Wearable sensors were found to be ineffective and impractical as the person may not feel comfortable wearing the sensor all the time, or they may forget to put it on. The PPI members expressed concerns as:

… *Well*,* I’ve seen my mother forgetting to wear her fall detection necklace…So is there something she won’t need to remember to wear and still alert if anything bad has happened*?

In addition, the PPI members also highlighted the need for privacy and not having the unobtrusive sensors look or behave like devices such as Amazon Echo, etc., which may cause distress for the person being monitored. They also mentioned that they would be uncomfortable using cameras in their bedrooms for privacy reasons. For example, a PPI member said:

…. *It would be good if you could consider people with different needs. For example*,* Alexa can be good for some*,* but its experience for people with dementia can be different and unpleasant*.

Another PPI member then added

*Yes*,* true! The same goes for privacy. I would not like cameras installed in my bedroom.*

Based on this feedback, the researchers decided to move ahead and develop Wi-Fi and radar-based unobtrusive sensors to monitor people’s respiration rate, hydration, and particular movements. The researchers also mentioned to PPI members that they would invite them to contribute again in the second stage, once the sensors have been developed.

### Phase II: developmental stage and the in-person PPI event

The physical demonstration of the sensing platforms and the sensors proved beneficial as it gave the public members an understanding of the sensors and their functionality, putting them in a position to participate in the discussion sessions with more clarity, as compared to presenting our work on slides only. The three sensing solutions were seen as valuable by the members of the public, who took a keen interest during the demonstration session. They asked questions and engaged in discussions during the demonstrations which helped them understand their capabilities in a better and clearer way.

### Feedback on the developed sensing solutions

In this session, feedback was taken on the strengths and concerns that the public members had for the sensors, the data being captured, and the parameters being measured. Moreover, suggestions were also taken for modifying the sensors to make them more impactful. The details of the responses are provided below.

#### Positive aspects of the sensing solutions

When asked about the positive aspects of the sensing solutions, the group highlighted that the sensors could be beneficial for health monitoring for younger generations and older adults. The group appreciated the parameters being measured and suggested that the sensors could help people manage their health in a proactive rather than reactive way if they could track deviations from an individual’s normal readings over time. A PPI member exclaimed

… *Wow! We are happy about what technology can do for care. This will especially be very useful to enhance the independence of older adults who do not want to leave their homes and go to care homes*….

Another PPI member added to this, saying

*These technologies aren’t just useful for people like us—they can benefit younger people too. If you can keep an eye on changes in your movement or breathing patterns, you can take action early and look after yourself before things get worse. It’s much better to catch issues early than to wait until something serious happens and you must go to the GP for a late diagnosis.….*

The group also liked how the sensors could be easily embedded in the home environment and furniture. They felt that the sensor devices were not too “alien” and felt “relatable” as they were part of their household (i.e., in furniture, walls, etc.). Moreover, they also liked the privacy-preserving aspect of the sensors as the data captured shows no images or videos. One of the PPI members claimed:

*“I would be happy to place the sensing solutions even in our bedrooms without any concerns of privacy invasion”.*

Another member of the group encouragingly stated:

*You can put these sensors in my house even tomorrow*,* I will not mind at all; they all are good!*

#### Concerns about the sensing solutions

The groups raised no major concerns regarding the sensing technologies. However, they were curious about the amount of data collected and whether the sensors would be active all the time. One PPI member highlighted a potentially complex scenario:

What happens if a guest visits me? Will my data be mixed with the guest? What if the guest objects to recording the data?

Another concern was regarding the interruption of Wi-Fi services within the home. PPI members also felt it was essential to consider inequalities and whether those without access to Wi-Fi would be excluded from using these devices. For example, a PPI members said

*In this situation*,* how would the data be collected*,* and how alerts would be sent if Wi-Fi access was interrupted*?

Another PPI member added to this, saying

*I hope you all are considering health inequalities*,* too. There are people who can’t afford or do not have a Wi-Fi connection*.

. The group also wanted to know

…. how will people be selected to use these technologies?

A PPI member suggested

*… It would be good if the NHS could provide these to the people and suggest the devices to those who need them the most*.

#### Suggestive modifications for additional parameter monitoring

When asked about the modifications that needed to be made to the sensing solutions, the public members were keen on providing feedback. They stated

… *Though all the sensors provide valuable information*,* the addition of blood pressure and temperature measurement along with breathing could add more value*.

The group suggested

*… The movement sensor should track changes in behaviour or the amount of activity to determine any deviation from normality within a day. This could also potentially help in the tracking and care of dementia patients*.

The suggestion for the hydration sensor was

… *It would be better if it could also detect the absorption level of an incontinence pad to alert a carer when a pad needs changing or whether the hydration sensor could monitor and alert a carer if someone has soiled the bed or chair*.

### Co-design of the sensing solutions for further improvements

Specific questions were designed for the PPI workshop to involve the public members in the future development of these sensing systems. The questions and discussion responses are detailed below.

#### Steps to be taken if parameters deviate from normal

Several insightful sequential points in response to this topic were provided by the PPI members. These are stated in the same sequence as provided by them:

“*If the mentioned parameters deviate from normal behaviour*.

i. *The sensors should know what is considered “normal” versus “not normal” for each person. A baseline should be set for every person or as a common threshold. A suggestion was made to involve occupational therapists or share data with them as they are involved with people living independently and hence*,* will have a better idea of the “baseline” and what pitfalls*,* degradation*,* etc. occur to the individual.*
ii. *Prioritise who should be informed if a change from normal is recorded. According to the public members*,

- *The first preference is that it should go to the patient/person being monitored if the issue is minor or if just a single parameter is not normal.*
- *If escalated (i.e.*,* multiple parameters are not normal)- an alert should be sent to carers*,* nurses or whoever is responsible for caring for the person.*

iii. *In the case of care homes*,* a monitoring station for staff in care homes to see changes in their residents would be useful.*
iv. *It is important to educate and describe to carers and the people under care*,* the protocol for what stage of abnormal data would lead to associated alerts. This would inform what care should be provided or intervention should be used.*
v. *Complimentary interventions could be designed for certain events. For example*,* mild dehydration would send a notification to the user to drink. There should also be specific alarms sent to alert carers during a sleep apnoea or for a fall event.”*

#### Further enhancing study design

The group highlighted the importance of getting volunteers and placing the sensors in their homes, as soon as possible to test the various scenarios. Although they couldn’t identify one single activity/scenario that should be focused on caring for older adults is a constantly changing and dynamic feat. However, the group did highlight the need for testing in a crowded environment (more than 2 people) to validate if the sensors could determine each person’s parameters individually and whether they could effectively identify if someone in the group was unwell. The group also suggested looking at the “day-to-day activities in care homes” to find activities and scenarios to use in future experiments.

### Feedback on the PPI event

At the end of the workshop, the PPI members filled out an evaluation form. This was done to gauge the effectiveness of the in-person hosted PPI event, the information shared, and the overall evaluation of the discussion participation. The questions and results are summarised in Table 2. The number in each box indicates the number of participants choosing that option as an answer. Though the number of participants was 8, however, the responses collected were from seven participants as one participant left after the focus group sessions due to other commitments.

**Table 2:** Feedback on the PPI workshop

	Questions Asked	Responses		
		Yes	Somewhat	No
1	Did you feel that the pre-workshop information was clear?	7	0	0
2	Did you feel you had the opportunity to speak during the table discussion?	7	0	0
3	Did you feel like your contributions were valued?	7	0	0
4	Has attending today’s workshop been useful to you?	7	0	0
		***Very***	***Somewhat***	***Not***
5	How informative was the day overall?	7	0	0
		***Too much***	***About right***	***Too little***
6	What did you think about the depth/detail?	0	*7*	*0*
7	If you would like to expand on any of the above, please do so here.	*Only 1 responded: found the whole concept amazing that it has been developed.*		
8	Thinking about today’s workshop as a whole, is there anything you would have liked to be included or changed?	*2 responded: No.* *4 didn’t respond.* *1 responded: Perhaps a live demonstration of sensors in action.*		

## Discussion

The PPI events organised at the ideation stage and then later to demonstrate the developed sensing solutions for evaluation and co-design were successful and impactful. This is established by public members’ range of suggestions, detailed feedback, and valuable ideas for the co-design of remote health monitoring solutions. This also serves as a testament that the involvement of the patients and public members living these experiences can provide valuable insights to improve and guide research and eventually result in rapid user acceptance of the developed technology solutions for healthcare. The major outcomes, findings and impact of involving PPI members are discussed below.

### Major outcomes and findings

#### Acceptability, usability and relevance

One of the main aims of the PPI workshop was to assess the acceptability, feasibility and relevance of the sensor systems being developed for remote health monitoring. The public members showed keen interest in the technology and its potential uses. They agreed that the sensors are useful as they can indicate early risk factors associated with hydration, movement, and breathing, allowing users to take proactive action, and assisting them to look after their health in a better way. This highlights how important the early PPI work at the ideation stage, shaped the technologies presented in the workshop which were then well received. According to the PPI members, early indications of risks can help in early treatment and interventions, avoiding deterioration in health and decreasing the possibility of hospitalisation. Besides the usefulness, public members were appreciative of the idea of how the sensors could seamlessly be integrated into the home environment. They stated that this made these sensors more acceptable, maintaining house aesthetics and making them feel comfortable and relatable when placed inside their home furniture.

#### Privacy

When presented with the data being collected by the sensors, the members of the public were pleased with the privacy-preserving aspect of the data as images or videos were not captured. Due to experience, the public members were well-versed with the various kinds of sensors and sensor technologies but said that they are reluctant to use cameras due to privacy invasion and wearables due to their intrusive nature. Hence, they were accepting of the radio-frequency non-contact sensors. It was also suggested that thermal cameras would be acceptable if they did not capture faces or other identifiable features such as clothing.

Attendees raised concerns about the amount of data being captured and were interested to know what would happen to the data when they have a visitor. The data being collected may not be a source of privacy invasion for the residents themselves but may be a concern for the guests. Similarly, another caveat raised was that of the nurses or carers who visit the older adults living independently. They raised a concern if the carer(s) objected to the use of sensors being placed in the house. This led to the discussion of the importance of education and informing both carers and people under care about how the technologies work and how data is stored/shared so that they can be acceptable.

#### Data sharing in case of alarms

Some members of the group did highlight a few concerns regarding the sharing of data. They mentioned that in the first instance, the data/alarm should go to the person being monitored so that “they know what is wrong and can take action first.” However, the group also mentioned that, depending on the level of cognitive abilities of the individual, an alarm may also need to be sent out to the person that they “nominate” as their carer (nurse, caregiver, friends, family, etc.). The group said that the decision to data sharing should depend on the data and its relevance. For example, if a fall occurred the immediate person to whom the alert should be sent is a neighbour or family member living nearby. Similarly, severe and prolonged abnormalities in respiration hydration and movement parameters should be shared with the General Practitioners (GPs).

#### Inter-organisational collaboration and knowledge sharing

Feedback gathered from the members of the public indicates that for these solutions to be truly effective and operational, inter-organisational collaborations and knowledge sharing are of significance. For example, if an individual is accepting of the sensor system, then the carer should also be accepting towards these solutions. This can only happen if everyone is aware of their usefulness and impact. Similarly, for the alarm system to work correctly and effectively the clinicians and care centres should be aware of them. A suggestion was also made that the National Health Services (NHS) should be involved. This will enable them to recommend these technologies to the people who need them the most and would also help in cost-effectiveness and ease of sensor setup.

### Co-design suggestions for further improvements

#### Functionality enhancement

Temperature and blood pressure were also considered vital parameters to be monitored by the PPI members. They said that temperature can help to assess hypothermia, especially during the cost-of-living crisis. They also highlighted the issue of whether the movement sensors would ‘track” the person around the house or change in activities. In some cases, having a “tracking” feature would be useful for people living with dementia, for example, to know where they are going and make sure they do not get lost. Another useful case discussed was the number of times people tend to use the toilet. This is also related to movement and hydration and can be an important risk factor for a potential fall. For the hydration sensor, they asked that a feature be included to monitor the wetness of clothes or cushions in case an elderly adult has either soiled or spilt liquid. An alarm should be raised to facilitate timely attention from the carer for changing or cleaning purposes.

#### Volunteer testing and involving occupational therapist in co-design

Similarly, suggestions for including occupational therapists, appropriate volunteers from care homes and validating sensors in living homes where more than one person lives (couples or families) are important. The suggestions above highlight how co-designing technological healthcare solutions creates value and ensures that the implemented research solutions are operationally effective.

### PPI workshop impact, future considerations and challenges

Based on the feedback and suggestions, several important aspects of change were noted that will shape the current research work. The sensors should be personalised, and the devices need to be calibrated to the individual being monitored. The sensors should be portable so they can be used outside or in another home if the individual is staying elsewhere for a short period, for example with relatives. It was also noted that the sensors must be self-contained so that people can’t easily change their settings or ‘fiddle’ with them. There was a suggestion that feedback from the sensors could go to a smartphone application, in a similar way to some home security cameras. However, it was noted that not everybody has Wi-Fi which would allow them to benefit from this. Additionally, alerts should have a sound and a flashing light to cater for people with different disabilities, and alerts should go to both the older adult and the care provider. There were also ethical concerns about the continuous consenting capacity of the device users. Another challenge highlighted by the public members was ensuring that the hydration sensors were washable as they are embedded in cushions and mattresses. Moreover, an alert could remind someone to ‘Drink water’, raise the alarm about falling, or make an emergency call if such an event occurs. Another suggestion for raising an alert could be if the data and inferences from the sensors are integrated with mobile applications. This study contributes to the growing recognition of PPI not only in evaluating the merit of new digital solutions but also as a critical mechanism for ensuring that healthcare innovations align with patient values and needs. Similarly, our findings emphasize the importance of stakeholder engagement throughout the design and implementation phases of healthcare technologies, particularly in adapting PPI strategies to the unique challenges of older adult care. Furthermore, the advocacy role of PPI in regulatory and adoption processes underscores its significance in bridging the gap between innovation and practical integration into healthcare systems. Together, these insights reinforce the essential nature of PPI in advancing healthcare innovation across diverse contexts.

### Impact of the PPI event from the public member point of view

The comprehensive feedback and suggestions for co-designing the technological solutions indicate the deep level of involvement of the PPI members. Table 2, quantitatively assesses the PPI event’s impact on the members. From the table, it is evident that all the members found the session informative and clear. Their suggestions and concerns were listened to, and their contributions were valued. The researchers planned the event’s activities and time duration with a consideration that (i) the objective is achieved, and (ii) the PPI members are not fatigued by the end of the workshop. Concluding the event, the public members expressed interest in attending future PPI workshops for these research projects. In the future, there is a plan to host more PPI workshops for the projects after incorporating the suggestions received in the current event.

### Limitations

The event’s limitations include the limited number of PPI members who could attend the in-person event. Moreover, the sample size was geographically constrained as these members were mostly residing in or near Edinburgh. Another limitation included the demonstration of the sensors. Due to limited time, the researchers could only show the instant working and measuring of health parameters rather than long-term measurement results. However, to address this, another PPI workshop is planned in a year or so. Moreover, only older adults participated in the in-person hosted PPI event, and the event didn’t include other stakeholders. However, the researchers have been keeping in contact with clinicians and other stakeholders at various stages of the research.

### Comparison with the literature

In the literature, there are research studies that report the impact of patient and public involvement and advocate for its significance [13, 18, 35, 36]. However, standard procedures and methods to maximise their impact are still scarce [13, 19]. There are some open questions. For example, the number of times and stages at which the PPI members should be involved in a research project. This may depend on the duration and scope of the project. Some studies have included various stakeholders at the early stages of the study [16, 18]. Another open question is how many PPI members are considered enough for their roles such as they are people with lived experiences or carers and family members of the patients for which the technology is being developed. Some have included 9 PPI members [19], and others have included more stakeholders including clinicians and business stakeholders as well [16, 18]. Systematic procedures are required to deliver impactful user-centred designs andensure the diversity and inclusion of the PPI members.

### Future work

There will be further opportunities for these individuals to be involved in developing the technology in the future. There is a plan to involve these and more members in another PPI event that will be hosted next year. The objective is to incorporate the suggestions and feedback from this PPI event to shape and inform the three research projects. The PPI members who attended the event expressed willingness to continue to be involved in the testing of the devices in their own homes.

## Electronic supplementary material

Below is the link to the electronic supplementary material.

Supplementary Material 1

Supplementary Material 2

Supplementary Material 3

Supplementary Material 4

Supplementary Material 5

Supplementary Material 6

Supplementary Material 7

Supplementary Material 8

Supplementary Material 9

## Data Availability

Data sharing is not applicable to this article as no datasets were generated or analysed during the current study.

## Declarations

### GRIPP2 reporting guidelines

The PPI event has been reported according to the guidelines specified by GRIPP2 (Guidance for Reporting Involvement of Patients and the Public) long form [32]. This is an international consensus for the agreed checklist while reporting a PPI study. The information presented in this paper to map the requirements and checklist for GRIPP2 long form is provided in the supplementary material (Appendix 1).
