# Supplementary material for: Patient and public involvement in the co-design and assessment of unobtrusive sensing technologies for care at home: a user-centric design approach
Source: BMC Geriatr. 2025 Jan 21;25:48. doi: 10.1186/s12877-024-05674-y (PMC11749497; doi:10.1186/s12877-024-05674-y)
Supplement: Supplementary file 2 — Supplementary Material 2 [file 12877_2024_5674_MOESM2_ESM.pdf]

Appendix 1

Table 1- GRIPP LONG FORM

Section and Topic	Item	Page
**Section 1: Abstract of paper**		
1a: Aim	Report the aim of the study	1
1b: Methods	Describe the methods used by which patients and the public were involved	1
1c: Results	Report the impacts and outcomes of PPI in the study	2
1d: Conclusions	Summarise the main conclusions of the study	2
1e: Keywords	Include PPI, “patient and public involvement,” or alternative terms as keywords	2
**Section 2: Background to paper**		
2a: Definition	Report the definition of PPI used in the study and how it links to comparable studies	3
2b: Theoretical underpinnings	Report the theoretical rationale and any theoretical influences relating to PPI in the study	3,4
2c: Concepts and theory development	Report any conceptual models or influences used in the study	n/a
**Section 3: Aims of paper**		
3: Aim	Report the aim of the study	4
**Section 4: Methods of paper**		
4a: Design	Provide a clear description of methods by which patients and the public were involved	5-10
4b: People involved	Provide a description of patients, carers, and the public involved with the PPI activity in the study	5
4c: Stages of involvement	Report on how PPI is used at different stages of the study	5-10
4d: Level or nature of involvement	Report the level or nature of PPI used at various stages of the study	7,8
**Section 5: Capture or measurement of PPI impact**		
5a: Qualitative evidence of impact	If applicable, report the methods used to qualitatively explore the impact of PPI in the study	13-16
5b: Quantitative evidence of impact	If applicable, report the methods used to quantitatively measure or assess the impact of PPI	16
5c: Robustness of measure	If applicable, report the rigour of the method used to capture or measure the impact of PPI	n/a
**Section 6: Economic assessment**		
6: Economic assessment	If applicable, report the method used for an economic assessment of PPI	n/a
**Section 7: Study results**		
7a: Outcomes of PPI	Report the results of PPI in the study, including both positive and negative outcomes	11-16

7b: Impacts of PPI	Report the positive and negative impacts that PPI has had on the research, the individuals involved (including patients and researchers), and wider impacts	7-10
7c: Context of PPI	Report the influence of any contextual factors that enabled or hindered the process or impact of PPI	11,14
7d: Process of PPI	Report the influence of any process factors, that enabled or hindered the impact of PPI	21
7ei: Theory development	Report any conceptual or theoretical development in PPI that have emerged	n/a
7eii: Theory development	Report testing of theoretical models, if any	n/a
7f: Measurement	If applicable, report all aspects of instrument development and testing (eg, validity, reliability, feasibility, acceptability, responsiveness, interpretability, appropriateness, precision)	n/a
7g: Economic assessment	Report any information on the costs or benefit of PPI	n/a
**Section 8: Discussion and conclusions**		
8a: Outcomes	Comment on how PPI influenced the study overall. Describe positive and negative effects	17
8b: Impacts	Comment on the different impacts of PPI identified in this study and how they contribute to new knowledge	17-20
8c: Definition	Comment on the definition of PPI used (reported in the Background section) and whether or not you would suggest any changes	3,17-18
8d: Theoretical underpinnings	Comment on any way your study adds to the theoretical development of PPI	20,21
8e: Context	Comment on how context factors influenced PPI in the study	15
8f: Process	Comment on how process factors influenced PPI in the study	18
8g: Measurement and capture of PPI impact	If applicable, comment on how well PPI impact was evaluated or measured in the study	17-21
8h: Economic assessment	If applicable, discuss any aspects of the economic cost or benefit of PPI, particularly any suggestions for future economic modelling.	n/a
8i: Reflections/critical perspective	Comment critically on the study, reflecting on the things that went well and those that did not, so that others can learn from this study	20,21
