# Supplementary material for: Patient and public involvement in the co-design and assessment of unobtrusive sensing technologies for care at home: a user-centric design approach
Source: BMC Geriatr. 2025 Jan 21;25:48. doi: 10.1186/s12877-024-05674-y (PMC11749497; doi:10.1186/s12877-024-05674-y)
Supplement: Supplementary file 4 — Supplementary Material 4 [file 12877_2024_5674_MOESM4_ESM.pdf]

Using sensors to monitor health in the home

Background

Care in later life for people in the community is typically low-tech and data-poor. We are looking to change this by developing new technologies that can be used to care for older adults in their own homes or in supported care environments. One piece of technology that we're looking at is a sensor platform that can be set up in the home to measure different aspects of health, such as breathing rate, hydration levels or falls.

We want this new technology to be developed with members of the public to make sure that it is looking at the most important challenges of ageing in the home. We also want to make sure that it is sharing information with the people who would be in the best position to provide support. This could be partner, close family member or carer.

How can I get involved?

We would like to speak to older adults and those who care for older adults to hear your thoughts on the technology. For example, what challenges of ageing in the home should we be focusing on? Is it important to monitor hydration levels, and is this something that is difficult to manage at the moment? What else would you like technology to support you with? How should this technology alert someone to say that support is needed?

What is the time commitment?

We would like to invite older adults and informal carers to join us at the Alrick building at the King's Buildings campus in Edinburgh on **Wednesday 9th August 2-4pm**. During the 2-hour session, you will have the opportunity to test out the sensors and discuss how we could use them to support carers or older adults living at home with aspects of care. We will also discuss how we could maximise the functionality of the sensors through an alert system.

Payment and expenses

You will receive £50 in vouchers as a thank you for joining this session. The nearest bus stops are located on Mayfield Road approximately 160 metres from the Alrick Building. Persons with mobility difficulties arriving by vehicle can access Alrick Building from Gate 3 or 4 which are located on Mayfield Road. There is also limited parking available on the campus. We can reimburse your travel expenses, and you will need to send us your car registration number in advance if you choose to drive.

How will I be supported?

Jenny Sharma (Patient and Public Involvement and Engagement Co-ordinator at the ACRC) will be available to answer questions and will support you throughout. We will be joined by Imran Saied and Ricardo Contreras who are both working to develop technology to be used in the home.

What should I do if I'm interested?

If you would like to register your interest in this workshop, please email jenny.sharma@ed.ac.uk by **Monday 3rd July**. We will then contact you with further details.

Privacy statement

The information that you provide will be used by the Advanced Care Research Centre (ACRC) to process your involvement and communicate with you about ACRC involvement in research activities.

BioCube1, Edinburgh BioQuarter
9a Little France Road
Edinburgh EH16 4UX
ACRC@ed.ac.uk
www.edin.care

The University of Edinburgh is a charitable body, registered in Scotland, with registration number SC005336.

We are using information about you because you have given us your consent to do so.

We will hold the personal data you have provided until you withdraw your consent for us to process your personal data. Your contact details will not passed to any researchers unless we have specific permission from you and you have indicated that you would like a researcher to contact you in relation to a project. We do not use profiling or automated decision-making processes.

If you have any questions, please contact Jenny Sharma, Patient and Public Involvement and Engagement Co-ordinator, jenny.sharma@ed.ac.uk.

This privacy statement is continued:
Continued privacy statement
