# Supplementary material for: Patient and public involvement in the co-design and assessment of unobtrusive sensing technologies for care at home: a user-centric design approach
Source: BMC Geriatr. 2025 Jan 21;25:48. doi: 10.1186/s12877-024-05674-y (PMC11749497; doi:10.1186/s12877-024-05674-y)
Supplement: Supplementary file 5 — Supplementary Material 5 [file 12877_2024_5674_MOESM5_ESM.pdf]

Evaluation form

We would appreciate your feedback on today's workshop. Please take a couple of minutes to answer the questions below.

	Yes	Somewhat	No
Did you feel the pre-workshop information was clear?	✓		
Did you feel that you had the opportunity to speak during the table discussions?	✓		
Did you feel like your contributions were valued?	✓		
How informative was the day overall? (please circle)	**Very** / somewhat / not informative		
What did you think of the depth/detail? (please circle)	Too little / **about right** / too much		

1) If you would like to expand on any of the above, please do so here:

2) Has attending today's workshop been useful to you?

Yes.

3) Thinking about today's workshop as a whole, is there anything you would have liked included or changed?

Evaluation form

We would appreciate your feedback on today's workshop. Please take a couple of minutes to answer the questions below.

	Yes	Somewhat	No
Did you feel the pre-workshop information was clear?	✓		
Did you feel that you had the opportunity to speak during the table discussions?	✓		
Did you feel like your contributions were valued?	✓		
How informative was the day overall? (please circle)	Very / somewhat / not informative		
What did you think of the depth/detail? (please circle)	Too little / about right / too much		

1) If you would like to expand on any of the above, please do so here:

2) Has attending today's workshop been useful to you?

✓

3) Thinking about today's workshop as a whole, is there anything you would have liked included or changed?

No.

Evaluation form

We would appreciate your feedback on today's workshop. Please take a couple of minutes to answer the questions below.

	Yes	Somewhat	No
Did you feel the pre-workshop information was clear?	✓		
Did you feel that you had the opportunity to speak during the table discussions?	✓		
Did you feel like your contributions were valued?	✓		
How informative was the day overall? (please circle)	Very / somewhat / not informative		
What did you think of the depth/detail? (please circle)	Too little / about right / too much		

1) If you would like to expand on any of the above, please do so here:

Found the whole concept amazing. That it has been developed

2) Has attending today's workshop been useful to you?

Yes - very interesting.

3) Thinking about today's workshop as a whole, is there anything you would have liked included or changed?

No

Evaluation form

We would appreciate your feedback on today's workshop. Please take a couple of minutes to answer the questions below.

	Yes	Somewhat	No
Did you feel the pre-workshop information was clear?	✓		
Did you feel that you had the opportunity to speak during the table discussions?	✓		
Did you feel like your contributions were valued?	✓		
How informative was the day overall? (please circle)	Very / somewhat / not informative		
What did you think of the depth/detail? (please circle)	Too little / about right / too much		

1) If you would like to expand on any of the above, please do so here:

2) Has attending today's workshop been useful to you?

Yes, its very interesting and possibly very useful.

3) Thinking about today's workshop as a whole, is there anything you would have liked included or changed?

Evaluation form

We would appreciate your feedback on today's workshop. Please take a couple of minutes to answer the questions below.

	Yes	Somewhat	No
Did you feel the pre-workshop information was clear?	✓		
Did you feel that you had the opportunity to speak during the table discussions?	✓		
Did you feel like your contributions were valued?	✓		
How informative was the day overall? (please circle)	Very / somewhat / not informative (circled: Very)		
What did you think of the depth/detail? (please circle)	Too little / about right / too much (circled: about right)		

1) If you would like to expand on any of the above, please do so here:

2) Has attending today's workshop been useful to you?

Yes.

3) Thinking about today's workshop as a whole, is there anything you would have liked included or changed?

Perhaps a live demonstration of sensors in action.

Evaluation form

We would appreciate your feedback on today's workshop. Please take a couple of minutes to answer the questions below.

	Yes	Somewhat	No
Did you feel the pre-workshop information was clear?	✓		
Did you feel that you had the opportunity to speak during the table discussions?	✓		
Did you feel like your contributions were valued?	✓		
How informative was the day overall? (please circle)	Very / somewhat / not informative		
What did you think of the depth/detail? (please circle)	Too little / about right / too much		

1) If you would like to expand on any of the above, please do so here:

2) Has attending today's workshop been useful to you?

Very useful & interesting

3) Thinking about today's workshop as a whole, is there anything you would have liked included or changed?

Evaluation form

We would appreciate your feedback on today's workshop. Please take a couple of minutes to answer the questions below.

	Yes	Somewhat	No
Did you feel the pre-workshop information was clear?	✓		
Did you feel that you had the opportunity to speak during the table discussions?	✓		
Did you feel like your contributions were valued?	✓		
How informative was the day overall? (please circle)	Very / somewhat / not informative		
What did you think of the depth/detail? (please circle)	Too little / about right / too much		

1) If you would like to expand on any of the above, please do so here:

2) Has attending today's workshop been useful to you?

Yes

3) Thinking about today's workshop as a whole, is there anything you would have liked included or changed?
