# Supplementary material for: Patient and public involvement in the co-design and assessment of unobtrusive sensing technologies for care at home: a user-centric design approach
Source: BMC Geriatr. 2025 Jan 21;25:48. doi: 10.1186/s12877-024-05674-y (PMC11749497; doi:10.1186/s12877-024-05674-y)
Supplement: Supplementary file 6 — Supplementary Material 6 [file 12877_2024_5674_MOESM6_ESM.pdf]

ACRC
Advanced Care Research Centre

Using sensors to monitor health in the home
Workshop details

Date: Wednesday 9th August 2023

Time: 2pm – 4pm
Refreshments will be available from 1:50pm so that we can start at 2pm

Location: Room GS 18, 4th floor
Alrick Building
Max Born Crescent
The King's Buildings
Edinburgh
EH9 3BF
(Use King's Buildings Entrance 4)

Directions

The workshop will be held in **Room GS 18** in the Alrick Building at **The King's Building campus.** This is part of the University of Edinburgh's School of Engineering.

Bus: The building is well serviced by buses from across the city, including Lothian Bus routes 24, 42, 67 that stop on Mayfield Road, and buses 38 and 41 that stop on West Mains Road.

Parking: If you would like to park on campus, please purchase a day pass from the parking office using the link below. You will need to make an account in order to do this. We will then refund you for your parking.

Parking permit link:
https://www.ed.ac.uk/transport/parking/visitors

Alternatively, free parking is available on the residential roads around the campus, such as on Ross Road, Hallhead Road and Esslemont Road.

Main entrance: The entrance to the Alrick Building is on Max Born Crescent. This is through Gate 4 on Mayfield Road. The building is then on your right hand side.

Click the map on below to get directions to The Alrick Building. Below the map is a picture of building as seen from the outside.

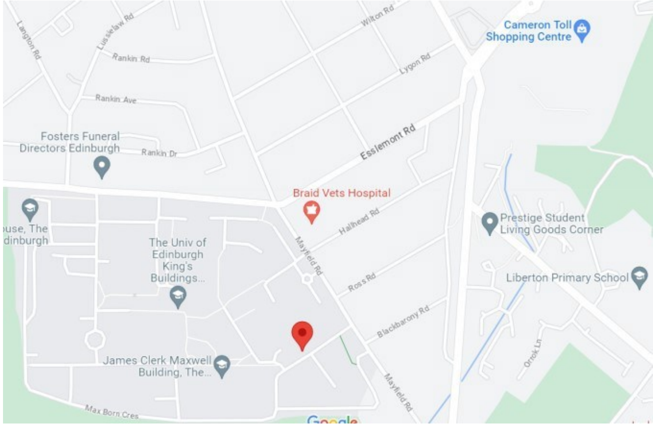

Location of the Alrick Building

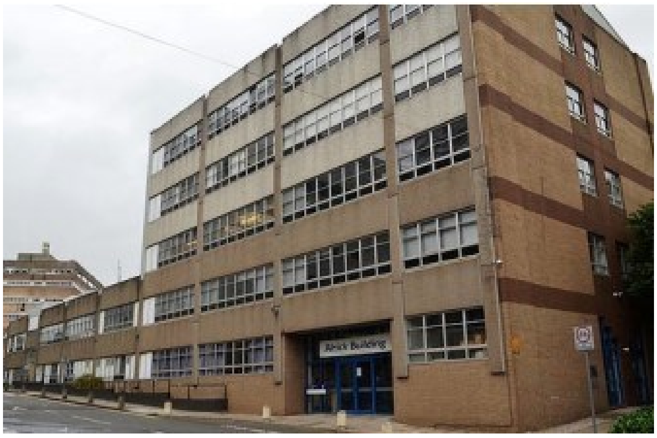

The Alrick Building from the outside

BioCube1, Edinburgh BioQuarter
9a Little France Road
Edinburgh EH16 4UX
aim-cisc@ed.ac.uk
www.edin.care

The University of Edinburgh is a charitable body, registered in Scotland, with registration number SC005336.

How to find Room GS 18

One of our team will meet you at the entrance to the building and direct you towards the meeting room. Room GS 18 is located on the 4th floor and has lift access. Another member of our team will meet you on the 4th floor to show you to the room.

Facilities and Accessibility:

There is no step access into the Alrick Building and Room GS 18 is accessible via a lift. Ladies toilets and an accessible toilet are located on the ground floor. The mens toilets are upstairs. There will be a break halfway through the workshop and light refreshments will be provided throughout.

If you need any additional requirements, questions, or difficulty finding the workshop, please don't hesitate to contact.

BioCube1, Edinburgh BioQuarter
9a Little France Road
Edinburgh EH16 4UX
aim-cisc@ed.ac.uk
www.edin.care

The University of Edinburgh is a charitable body, registered in Scotland, with registration number SC005336.
