# Supplementary material for: Patient and public involvement in the co-design and assessment of unobtrusive sensing technologies for care at home: a user-centric design approach
Source: BMC Geriatr. 2025 Jan 21;25:48. doi: 10.1186/s12877-024-05674-y (PMC11749497; doi:10.1186/s12877-024-05674-y)
Supplement: Supplementary file 7 — Supplementary Material 7 [file 12877_2024_5674_MOESM7_ESM.pdf]

Using sensors to monitor health in the home

9th August 2023

Jenny Sharma, Public Involvement Co-ordinator

Imran Saied, Research Associate

Longfei Chen, Research Associate

Nazia Gillani, PhD student

Aaesha Alzaabi, PhD student

ACRC

Advanced Care Research Centre

Housekeeping

- What to do if the fire alarm goes off
- Refreshment breaks
- Toilets
- Taking notes of key points
- Photos- please complete and return the consent form
- Please turn your mobile phone to silent
- We will make the slides available afterwards

ACRC
Advanced Care Research Centre

Programme for today

- Introductions
- Sensor demonstrations
- Table discussions
- Tea/ coffee break
- Table discussions
- Round up and close

Aims of today:

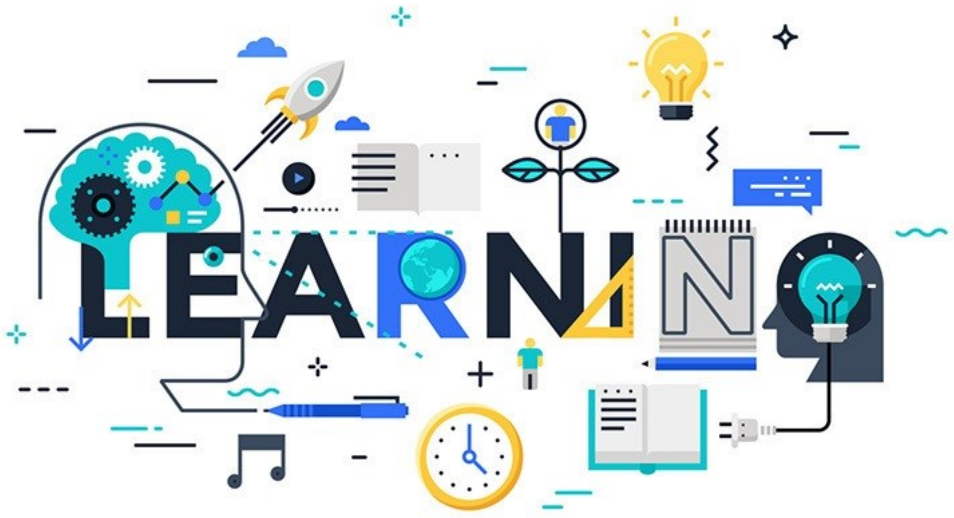

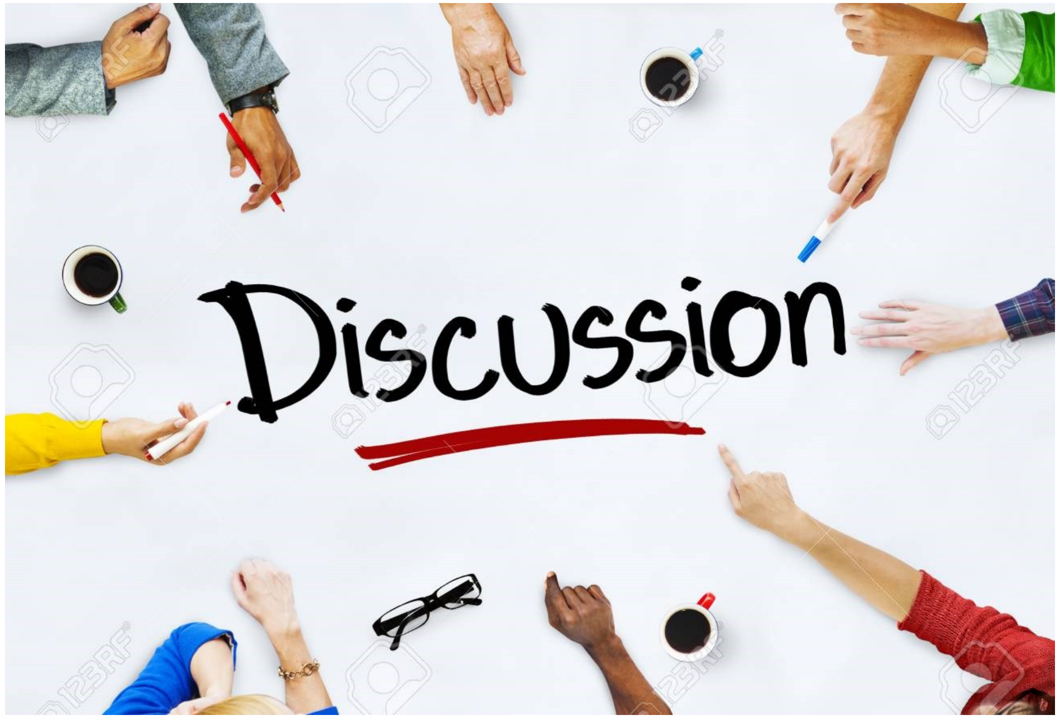

ACRC
Advanced Care Research Centre

Your voice is key

- Want to design our research **with** members of the community, not **for** people
- You bring lived experience and your professional experience
- There’s no correct answer
- Challenge us!

Introductions

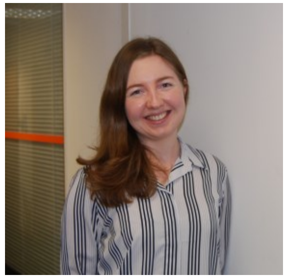

Jenny Sharma
Public Involvement Co-ordinator

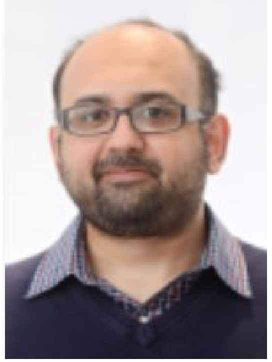

Dr Imran Saied
Postdoctoral Research Associate

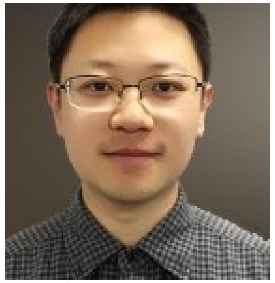

Dr Longfei Chen
Postdoctoral Research Associate

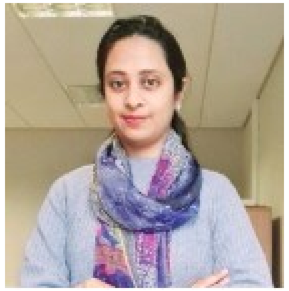

Nazia Gillani
PhD student

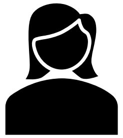

Aaesha Alzaabi
PhD student

THE UNIVERSITY of EDINBURGH

Introductions

- At your tables
  - Name
  - Why you were interested in joining today's workshop

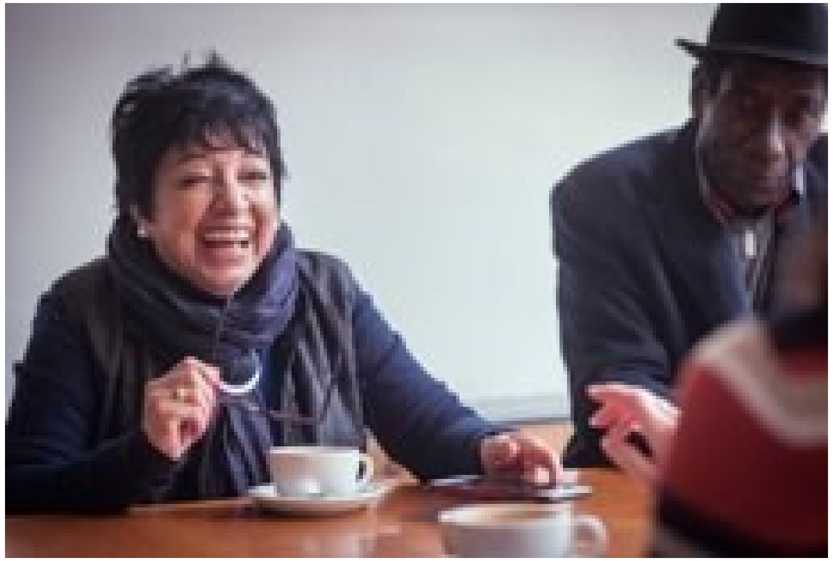

THE UNIVERSITY of EDINBURGH

ACRC
Advanced Care Research Centre

Background to the research and sensor demonstrations

Imran Saied

ACRC
Advanced Care Research Centre

Background of Unobtrusive Sensing Platform

- Our work -> developing unobtrusive sensors to monitor different physiological and falls parameters of people under care
  - Hydration
  - Gait/Activity
  - Breathing

- Our focus was to design the sensors to be integrated in household items (e.g., furniture, walls, etc)
  - Ensure they are unobtrusive and noninvasive.
  - Private and do not hinder day-to-day activities of people under care.
  - Safely monitor health parameters of individuals and enable their independence.

- Captured data from our sensors will then be processed and used in “alert systems” that detect abnormal patterns and prompt caregivers when care is needed.

THE UNIVERSITY of EDINBURGH

ACRC
Advanced Care Research Centre

Vision of Unobtrusive Sensing Platform

June 2021 (Start)

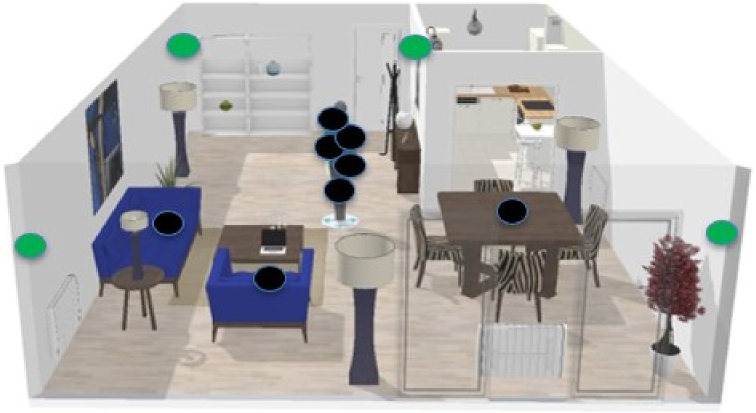

August 2023

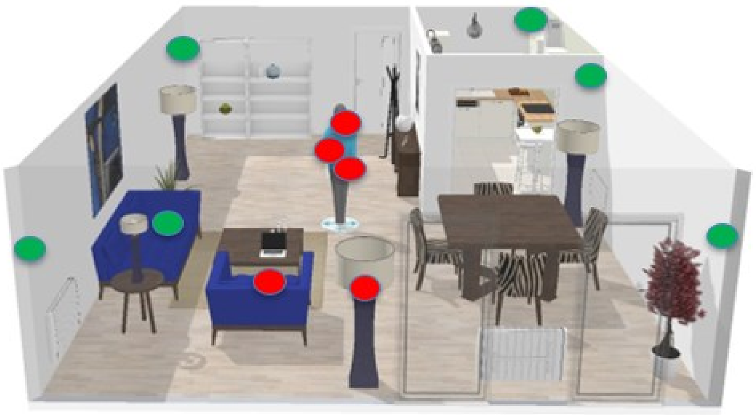

December 2023

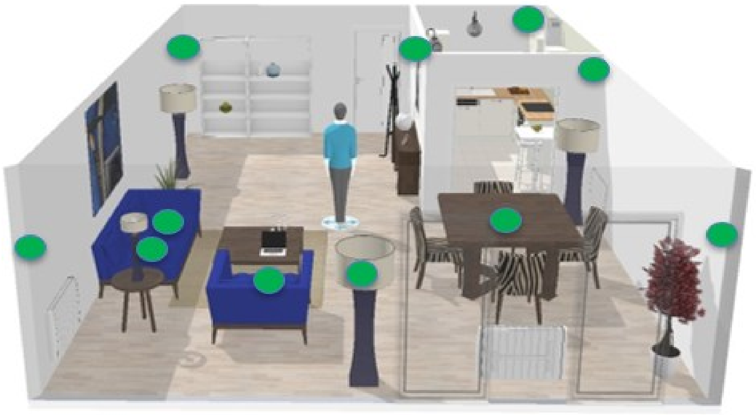

- Sensors (Contact/Wearable)
- Optimisation of Sensor
- Completely Unobtrusive Sensor

Aim -> Completely unobtrusive sensor platform that can monitor physiological/falls parameters.
Hybrid approach -> consisting of a good mix of commercial and developed sensors.

THE UNIVERSITY of EDINBURGH

ACRC
Advanced Care Research Centre

Unobtrusive Hydration Monitoring Device

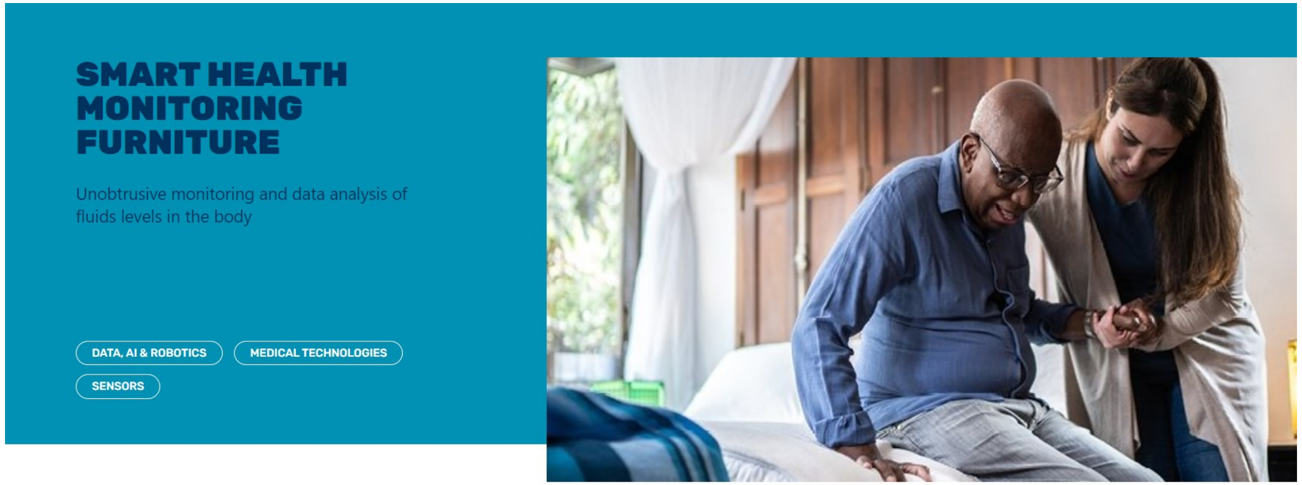

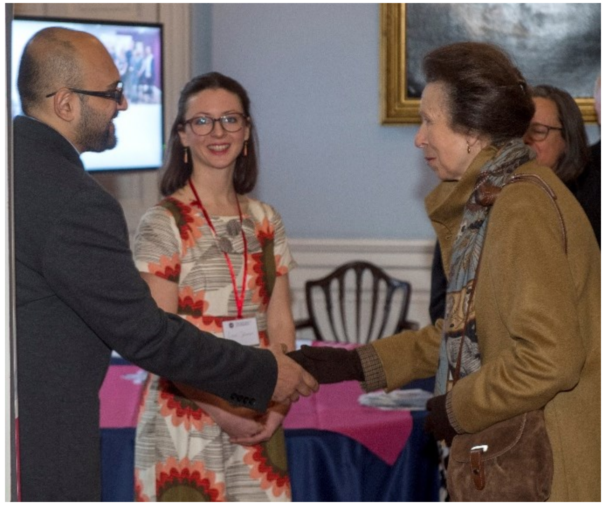

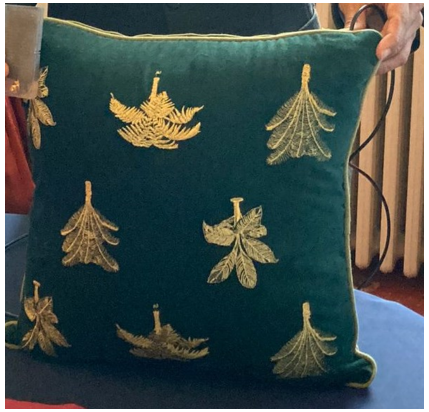

Edinburgh Innovations | Smart health monitoring furniture

- Currently, there is no way to unobtrusively monitor hydration of people.
- Important for people under care in hospitals and at home.
- Exciting new invention developed in our group (Patent filed).
- Sensors integrated inside furniture (cushions, mattress, etc) to unobtrusively monitor hydration levels of people.
- Help predict abnormal hydration events to enhance independent living (e.g., Dehydration, Urinary Incontinence, and Pneumonia/Pleural Effusion).
- Provides timely care and interventions based on fluid readings.
- Demoed to care home (Braid Health) and Chancellor of the University (HRH, The Princess Royal)

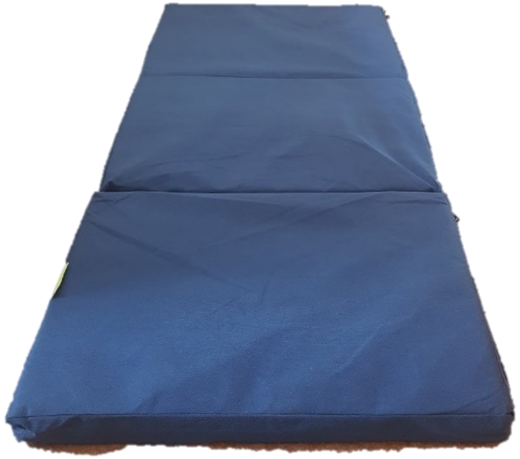

ACRC
Advanced Care Research Centre

Demo of the Hydration Monitoring Cushion

Monitoring Movement

I Am

Nazia Gillani - PhD Student

My Research Focus

Monitoring various movement patterns and specific parameters related to movements

It is Important because

The study of movement can help in

- the evaluation of our health status.
- highlighting any risk factors that may indicate a decline in our functional mobility

Clinical Implication

Timely assessment of such risk factors may help us in preventing adverse outcomes. One such example is ‘falls’.

Unobtrusive Activity/Movement Monitoring Sensor

One sensor covers the entire area of the room.

Sensor can see up to

- a max Distance of 9 m
- with wide angle area covered

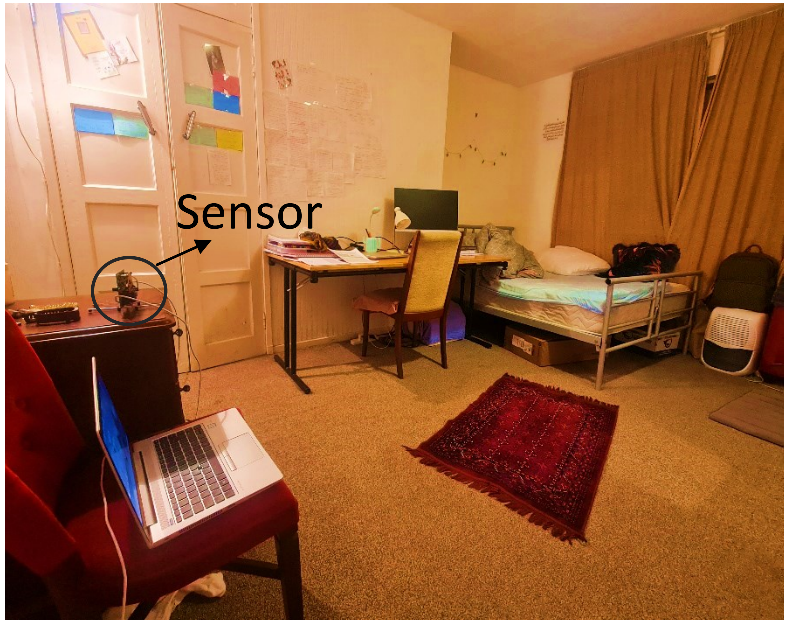

(Room: 3.7m x 2.5m)

ACRC
Advanced Care Research Centre

Unobtrusive Sensor – Falls/Movement

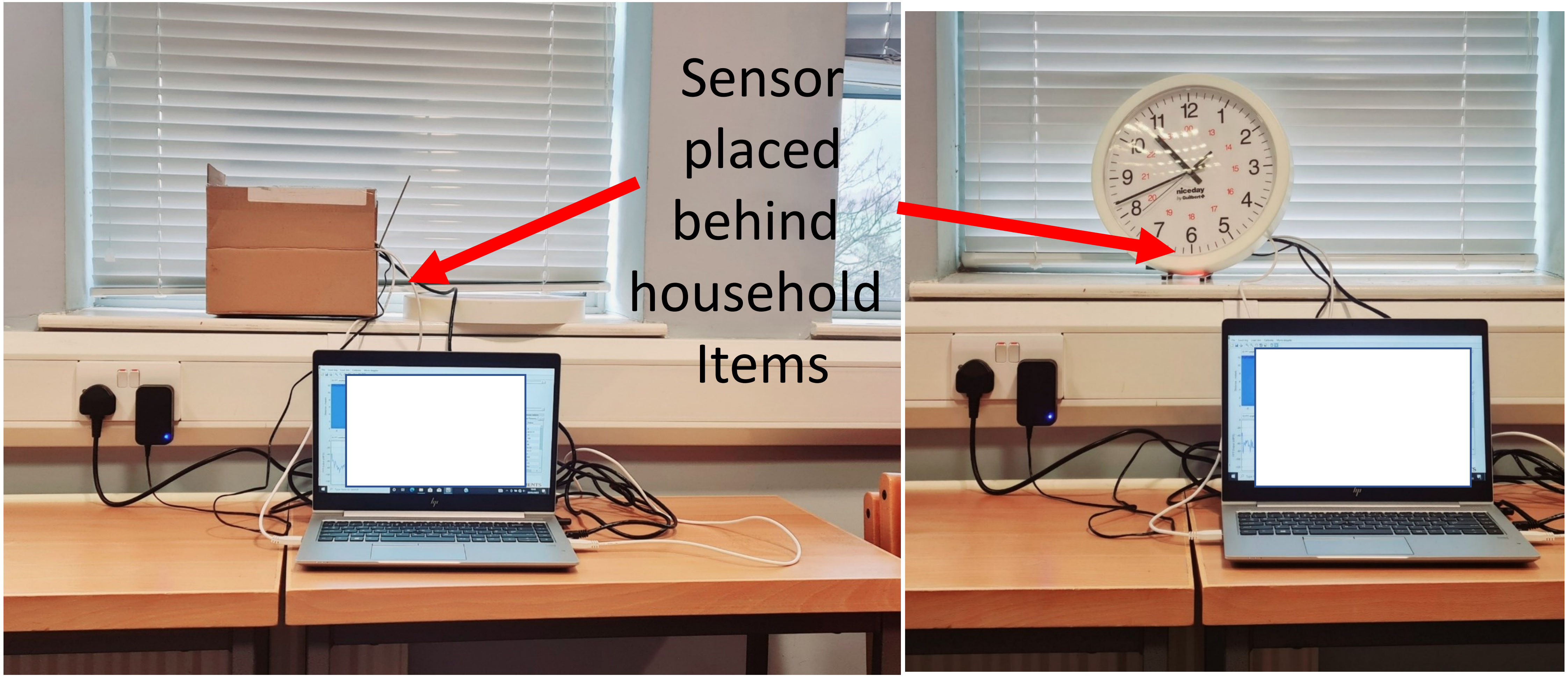

Advanced Care Research Centre

Unobtrusive Sensor System – Activity Monitoring (Updates)

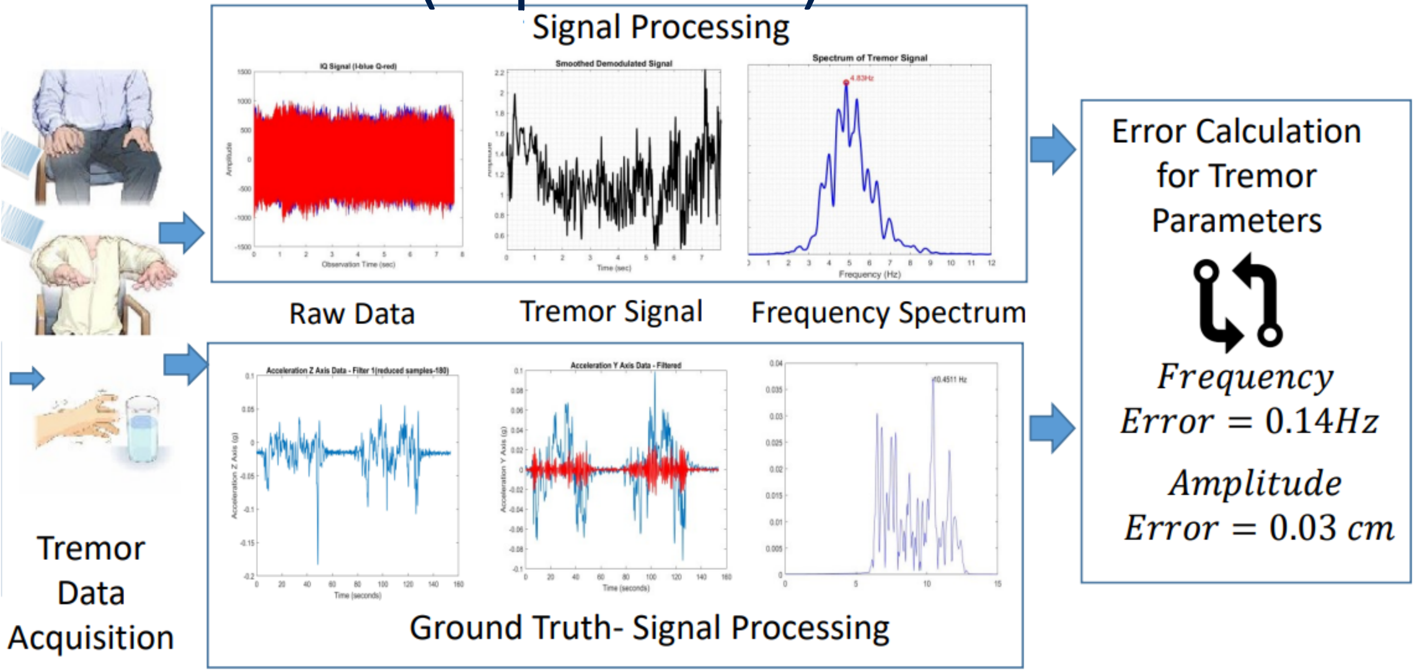

- Tremors are characteristic feature of progressive neurological diseases, such as essential tremor (ET) and Parkinson's disease (PD).
- For people aged between 40 and 60 years, the incidence rate is 4%–5%. This increases to 9% for people aged 60 years or more.
- Resulted validated by comparing data with method which is compatible with the FDA and the regulatory oversight

ACRC Advanced Care Research Centre

Activities in a Room

Motion detection in the room while:

a. Getting up from the bed, going to the washroom and returning.
b. Getting up from the bed and sitting on the chair.

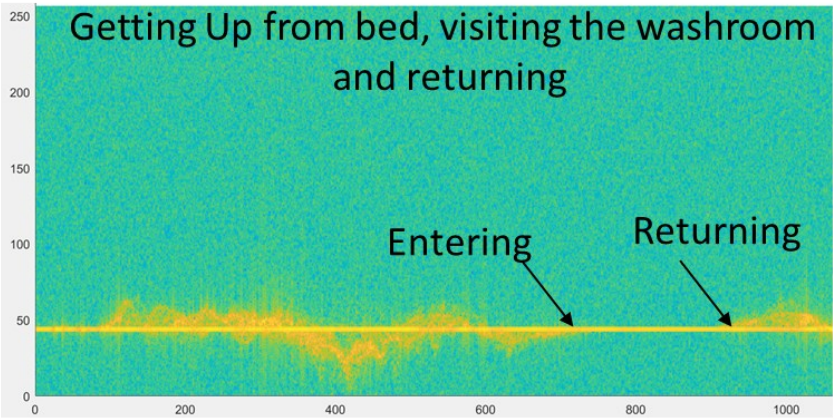

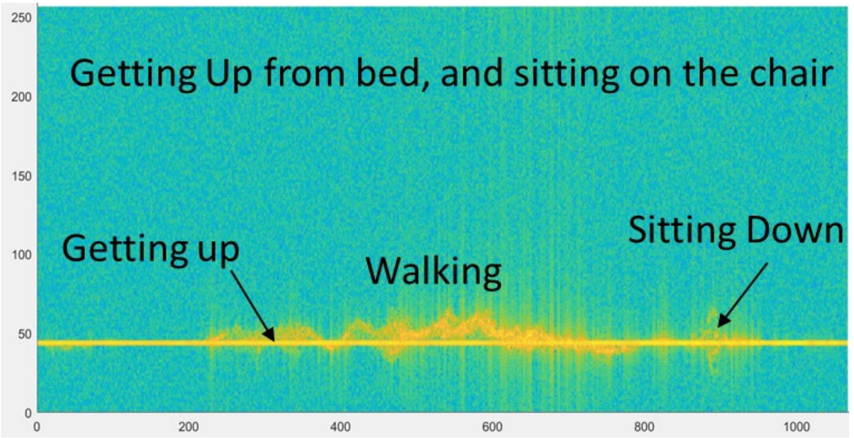

Advanced Care Research Centre

Activities in a Room

While sitting in the chair:

a. Reading a book/paper.
b. Sitting idle/watching TV.

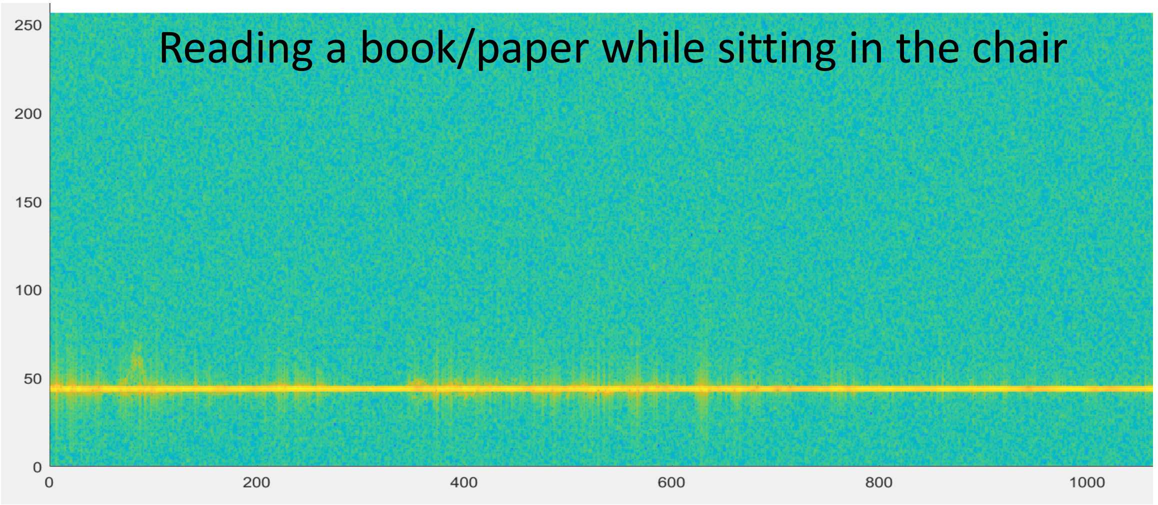

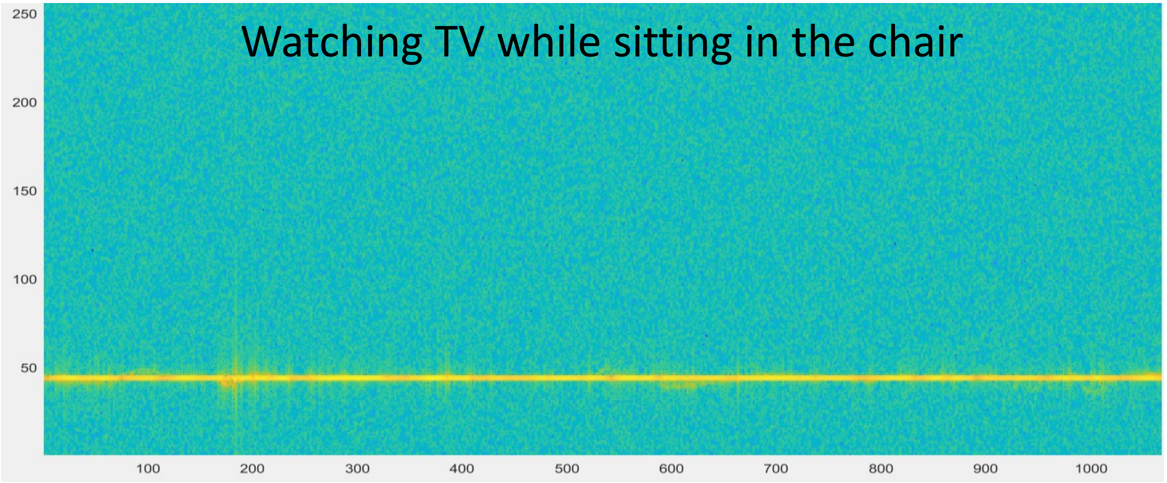

THE UNIVERSITY of EDINBURGH
ACRC
Advanced Care Research Centre

Demo of the Movement Monitoring Sensor

Wi-Fi Sensing

Unobtrusive health monitoring using RF sensors have been supported by the advancement in sensors and algorithms, enhancing applications such as respiration monitoring, heart rate monitoring, sleep staging, activities of daily life and falls recognition. In this work, we focus on respiration rate monitoring of the individual in a resting state (sitting).

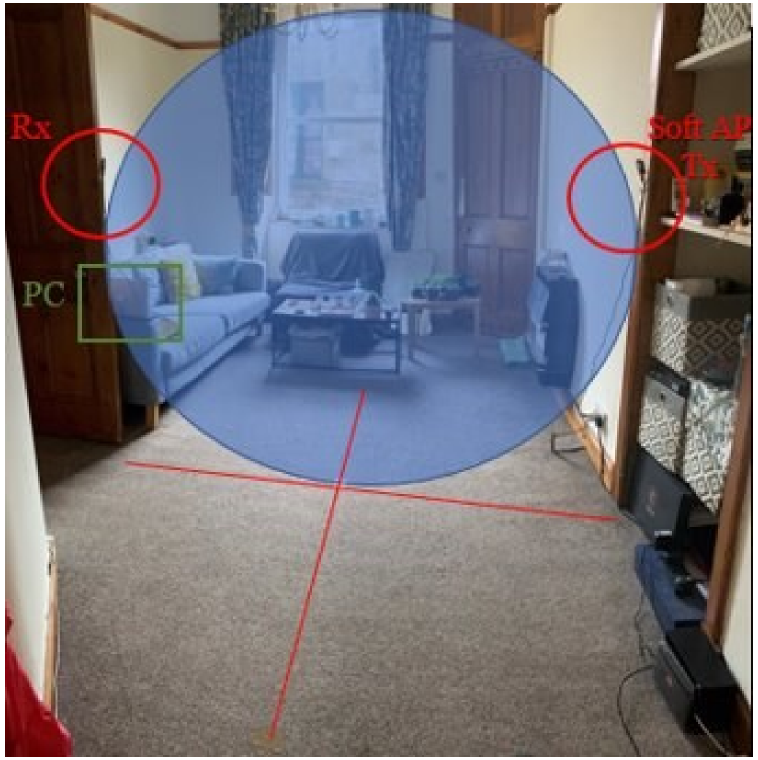

In-home monitoring scenario

THE UNIVERSITY of EDINBURGH
ACRC
Advanced Care Research Centre

Unobtrusive Breathing Monitoring Sensor

- Utilise Wi-Fi-based sensors to measure various breathing rates (e.g., slow, normal, and fast).
- Data from the sensors were compared with a medical-grade respiration belt to validate.
- Wi-Fi based sensors can be placed on the walls of a home and monitor breathing patterns of individuals while seated or lying down.

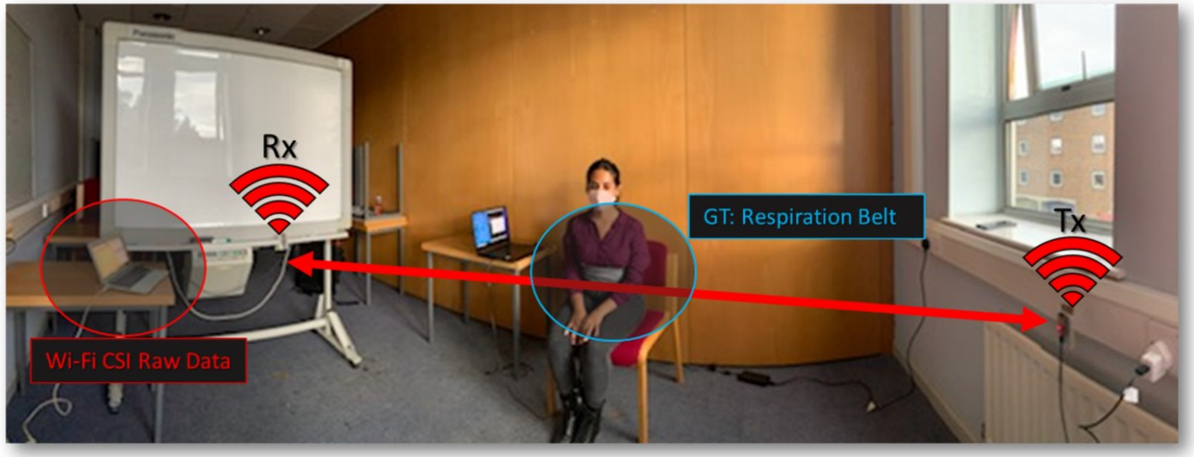

Figure. 1

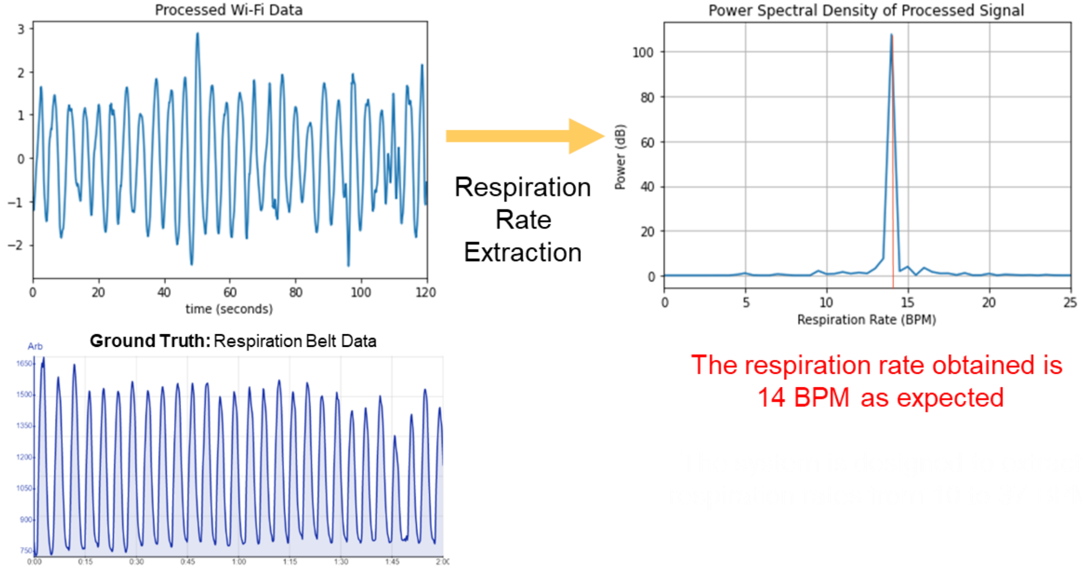

The respiration rate obtained is 14 BPM as expected

ACRC
Advanced Care Research Centre

Unobtrusive Breathing Monitoring Sensor – Sleep Apnoea Experiment

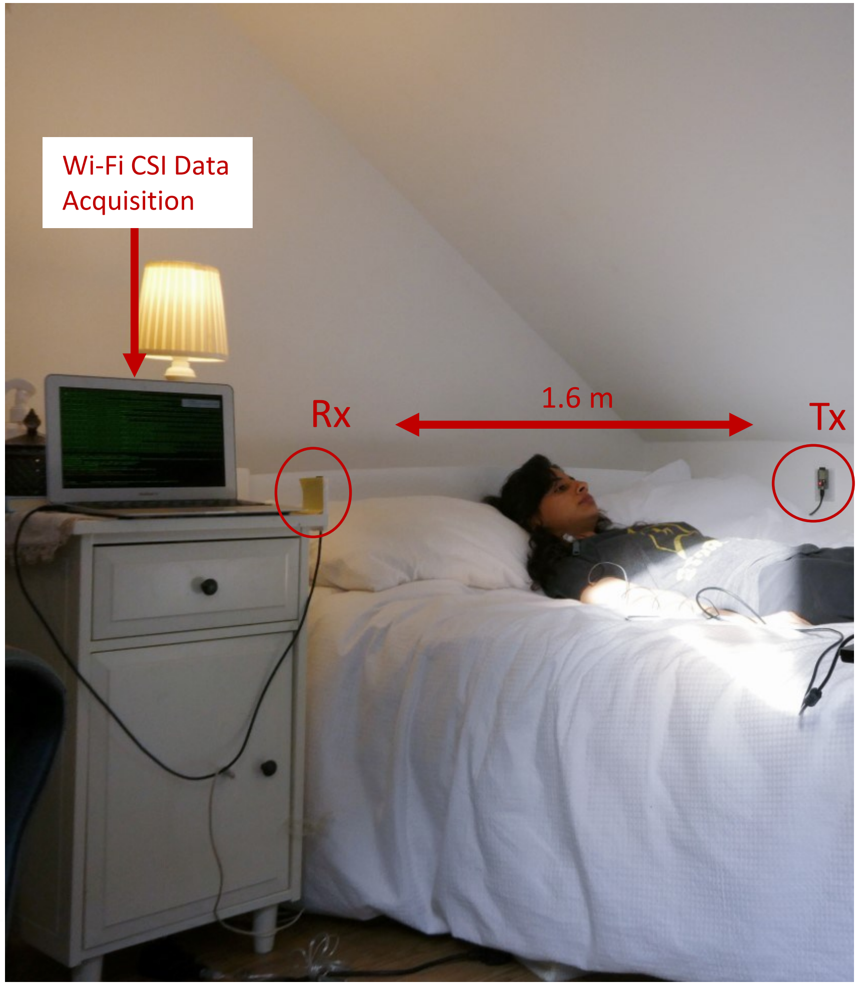

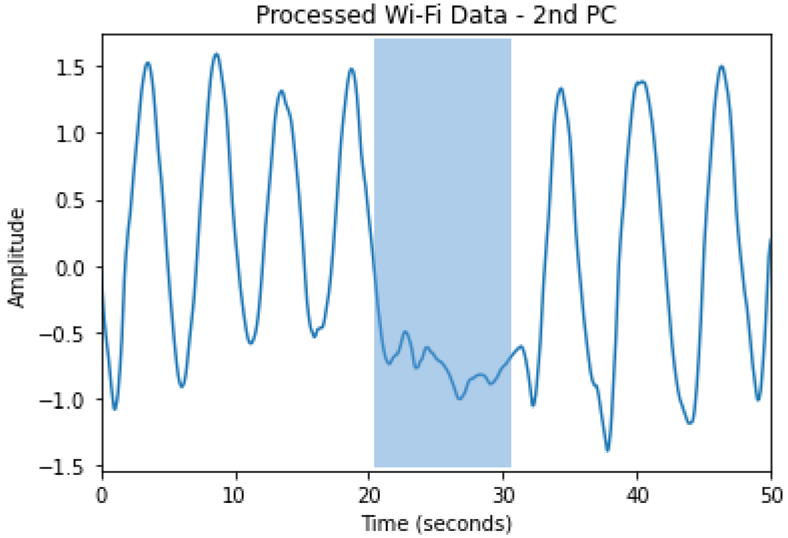

- Experiment was done to validate whether sensors could determine presence of sleep apnoea.
- Breathing was stopped for 10 seconds to simulate presence of sleep apnoea.
- Captured sensor data showed the breathing rate stopping and resuming

ACRC
Advanced Care Research Centre

Quick Demo of the Breathing Monitoring Sensor

THE UNIVERSITY of EDINBURGH

ACRC
Advanced Care Research Centre

Table discussions

1. How can we modify these sensors to make them more valuable to older adults and unpaid carers?

2. What aspects of the sensors do you particularly like?

3. What concerns do you have about the sensors?

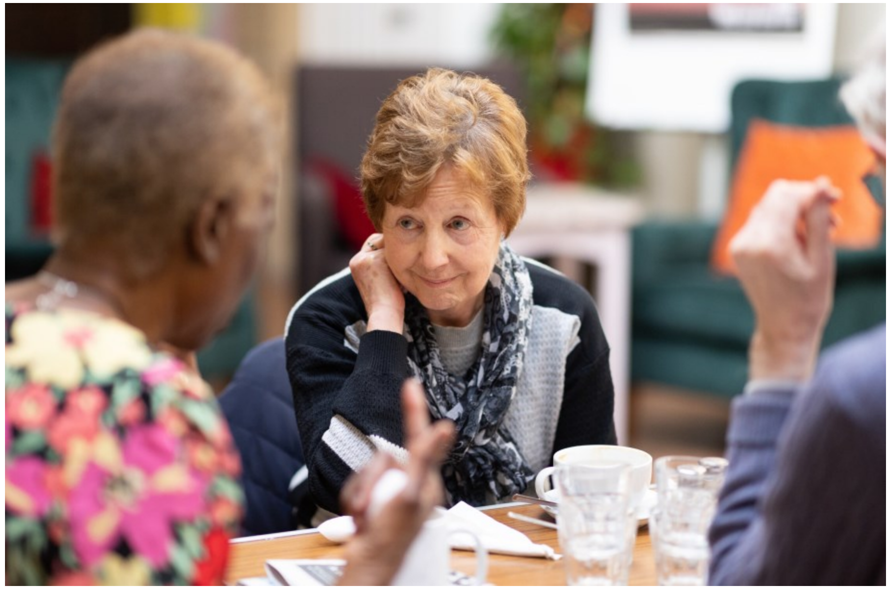

Break

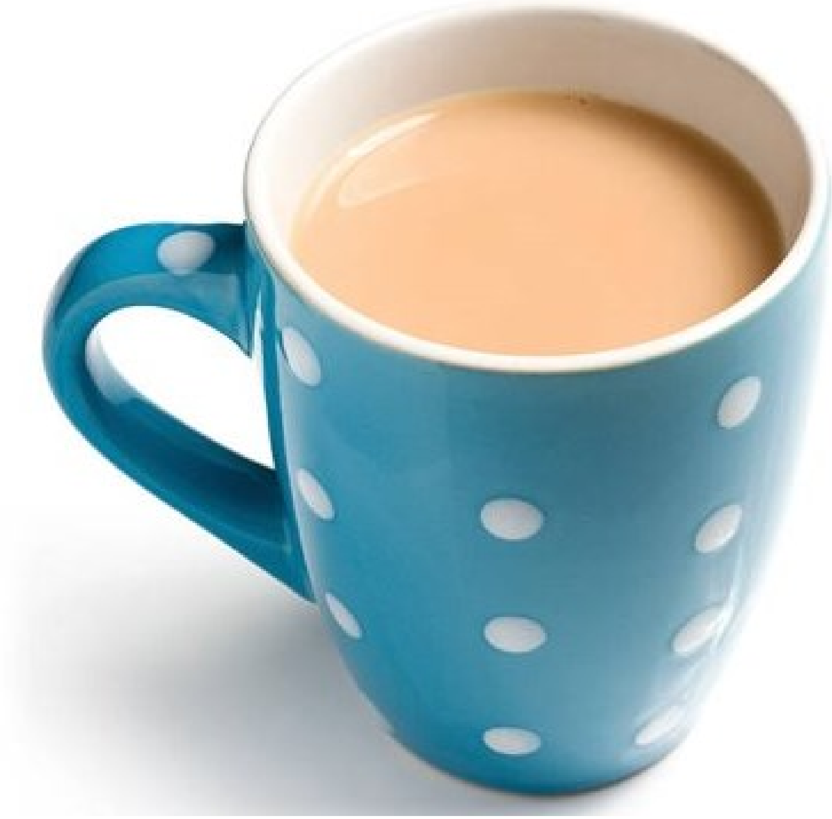

ACRC
Advanced Care Research Centre

Table discussions

1. What would you like the sensors to do if they record a change from normal hydration or walking pattern?

2. Are there any specific scenarios/ activities in everyday life during which these sensors would be particularly beneficial?

3. How else would you like to be involved in this work?

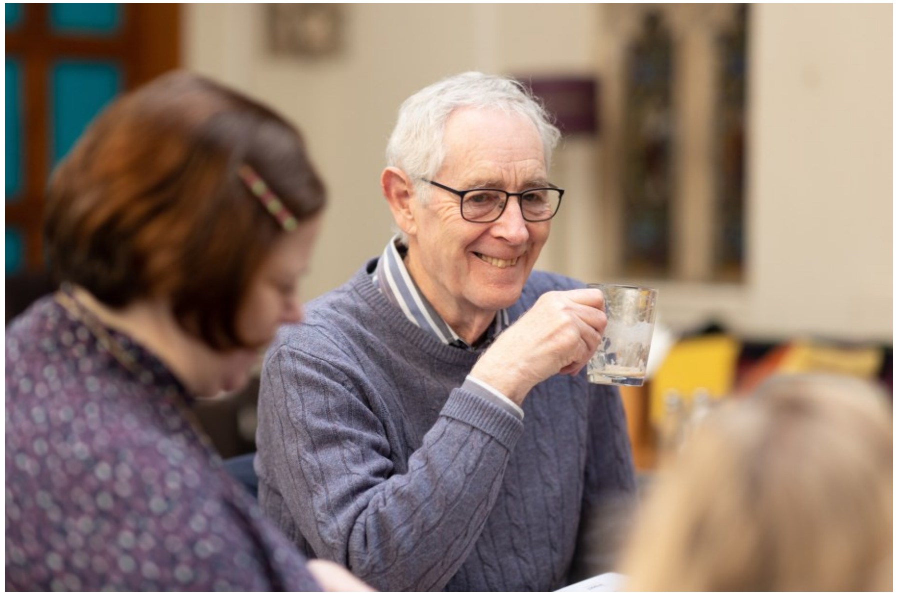

ACRC
Advanced Care Research Centre

Round up

Jenny Sharma

ACRC
Advanced Care Research Centre

Any final thoughts or comments?

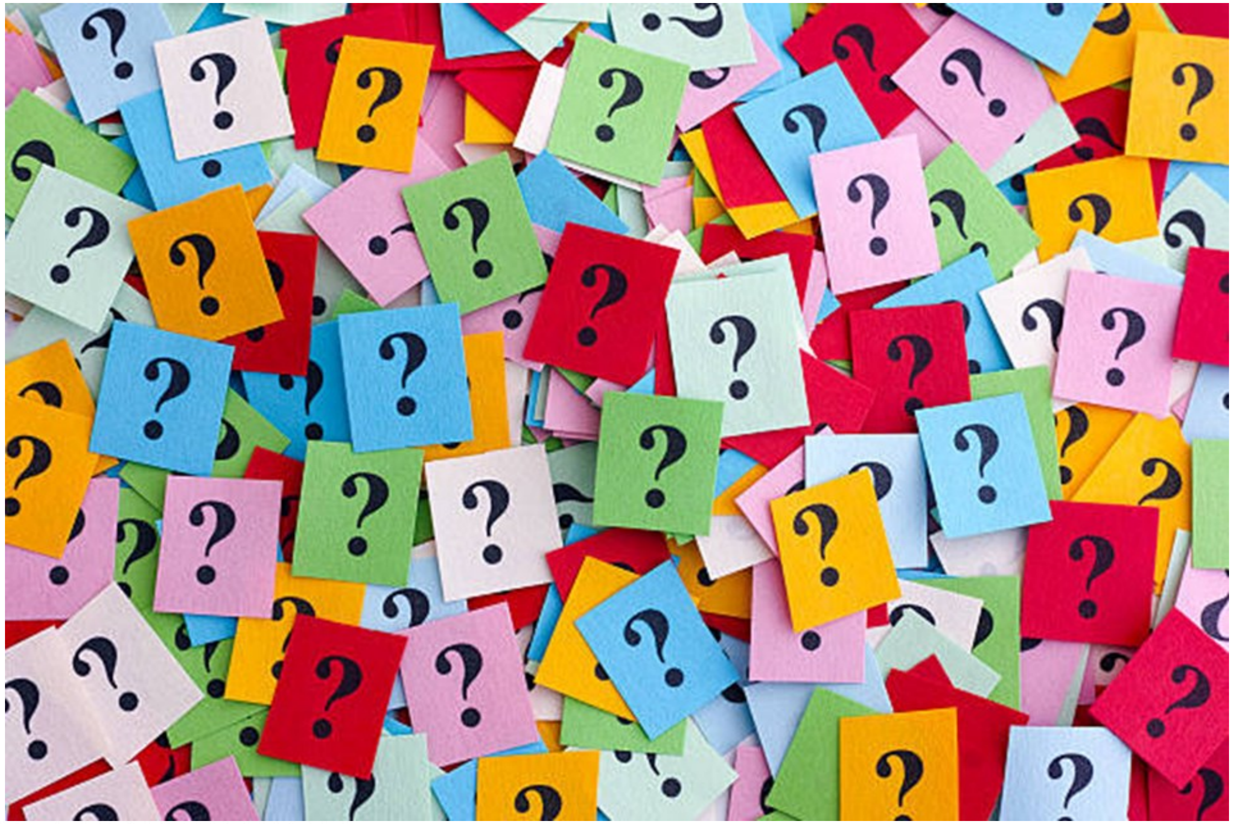

Advanced Care Research Centre

Round up

- Next steps:
  - Share today's key learnings with the team
  - Optimising the sensors (4-6 months)
  - Developing an alert system (4-6 months)
  - Academic paper on PPI work

- Opportunity to be involved again to test the sensors (early 2024)

- Feedback form

Thank you!

- Jenny: jenny.sharma@ed.ac.uk
- Imran: isaied@exseed.ed.ac.uk
- Longfei: longfei.chen@ed.ac.uk
- Nazia: N.Gillani@sms.ed.ac.uk
- Aaesha: A.H.M.Alzaabi@sms.ed.ac.uk

THE UNIVERSITY of EDINBURGH

ACRC
Advanced Care Research Centre
