# Supplementary material for: Patient and public involvement in the co-design and assessment of unobtrusive sensing technologies for care at home: a user-centric design approach
Source: BMC Geriatr. 2025 Jan 21;25:48. doi: 10.1186/s12877-024-05674-y (PMC11749497; doi:10.1186/s12877-024-05674-y)
Supplement: Supplementary file 8 — Supplementary Material 8 [file 12877_2024_5674_MOESM8_ESM.pdf]

Outlook

Fw: falls/frailty ....research

From Tughrul Arslan <Tughrul.Arslan@ed.ac.uk>
Date Tue 10/12/2024 13:02
To Nazia Gillani <N.Gillani@sms.ed.ac.uk>; Imran Saied <isaied@exseed.ed.ac.uk>

1 attachment (2 MB)
Copy of Tool-19-Falls-Data-Spreadsheet---Feb-2017-v2.xlsx;

From: Gordon, Jo <Jo.Gordon@nhslothian.scot.nhs.uk>
Sent: 19 March 2020 17:54
To: ARSLAN Tughrul <Tughrul.Arslan@ed.ac.uk>
Subject: RE: falls/frailty ....research

Hi Tughrul,

Attached is the falls data spreadsheet.

Apologies, I did not have the right address for your colleague Imran.

Thanks again,

Kind regards,

Jo.

From: Gordon, Jo
Sent: 19 March 2020 17:03
To: 'ARSLAN Tughrul'; 'imran.ahmed@ed.ac.uk'
Subject: RE: falls/frailty ....research

Dear Tughrul and Imran,

It was lovely to speak to you both today.

It was interesting to hear about the ACRC project and useful to have an initial discussion regarding possible joint working.

I like your ideas of a focus around falls- both sensing falls, and predicting falls.

At a later date I will write an email introducing you to Amanda Fox (LTCP Manager), Dr Carl Bickler (LTCP medical strategic lead and GP), Andy Jones (ATEC-24 lead for technology for EHSCP/Lothians) and Elizabeth Payne (Telehealth Lead, LTCP) however at the moment all emails are critical in nature due to COVID-19. It would also be useful to introduce you to a EHSCP care home lead, or experience care home manager nearer the time of project design. At the moment we are to avoid contact with care home managers. You may also be interested to know that EHSCP runs a SmartHome for showcasing technology to improve lives, primarily in

the community, that would be another useful link for you. I do have a catalogue of the technology currently available within the partnership if useful. It is quite a large file.

In the first instance here is the good practice resource I mentioned:
https://www.careinspectorate.com/index.php/care-news-online/9-professional/2737-falls-and-fractures
The tool I highlighted was Tool 19, in excel. It can take time to download however I imagine you have that covered! As I mentioned the first 3 tabs are important, and there are a couple of tips to getting the third tab to work properly to display all graphs I can share. In due course I can attach the tool, a worked example of the third tab, and also a screenshot of the data we have through tableau dashboard, relating to TRAK/BOXI data, for A&E attendances and unplanned hospital admissions relating to falls, with care home names removed.

The idea of wearable and remote sensing, both hardware and software solutions, sounds fantastic. It would certainly be useful to measure factors such as location, physical activity, height from the ground, acceleration, temperature, blood pressure, heart rate, hydration, continence, blood oxygen saturation levels (usually measured by a pulse oximeter on the finger) and other vital signs. There may be some roles for mobility equipment (walking sticks, crutches, frames and trolleys), or equipment used to lift fallen residents from the ground (see hoists such as by Arjo, and the brand Mangar which creates inflatables, for existing equipment). The topic of improving sensors such as mats or laser sensors for beds, chairs, floors and otherwise, and links to alarms, voice messages, and lighting that works at night when residents get up to the toilet is worth considering.

It was interesting to discuss the topic of consent as the majority of care home residents have a power of attorney. So any research project, within ethical considerations, would need to build in obtaining appropriate consent, and look to work with the NHS Lothian Cauldicott Guardian. Also any intervention would require manager and staff involvement and likely training/close working with both. Care homes are busy places but once you get them on board they are fantastic and there is the potential to make a real difference to lives and to jobs in the process.

For information around 4.6% of over-65's in Edinburgh live in care homes, however in 2019 20% of admissions to orthopaedics were care home residents. You can find information on predicted population size in different age groups in the EHSCP IJB (integraded joint board) strategic report.

In summary this all sounds really positive, and certainly sounds like improvement work with the potential to look forwards to following current COVID-19 measures.

I hope that helps your work in the short term! Lovely to connect, and thank you so much for your interest in working with me and my team.

Kind regards,

Jo.

Jo Gordon
Falls Co-ordinator | Care Homes Project | Long Term Conditions Programme
07980 733 761 not currently office based
jo.gordon@nhslothian.scot.nhs.uk
Twitter: @EdinburghHSCP
Please note I work on Mondays, Wednesday mornings and Fridays
Edinburgh Health and Social Care Partnership Working together for a caring, healthier, safer Edinburgh

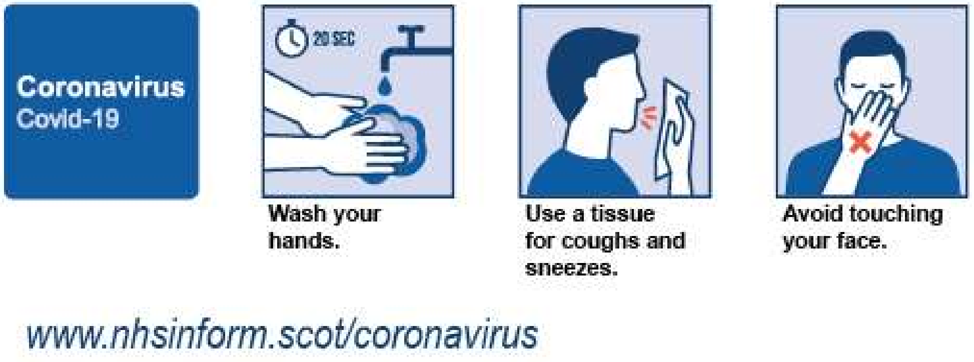

****
The information contained in this message may be confidential or legally privileged and is intended for the addressee only. If you have received this message in error or there are any problems please notify the originator immediately. The unauthorised use, disclosure, copying or alteration of this message is strictly forbidden.
****
