# Supplementary figures and images for: Patient and public involvement in the co-design and assessment of unobtrusive sensing technologies for care at home: a user-centric design approach
Source: BMC Geriatr. 2025 Jan 21;25:48. doi: 10.1186/s12877-024-05674-y (PMC11749497; doi:10.1186/s12877-024-05674-y)

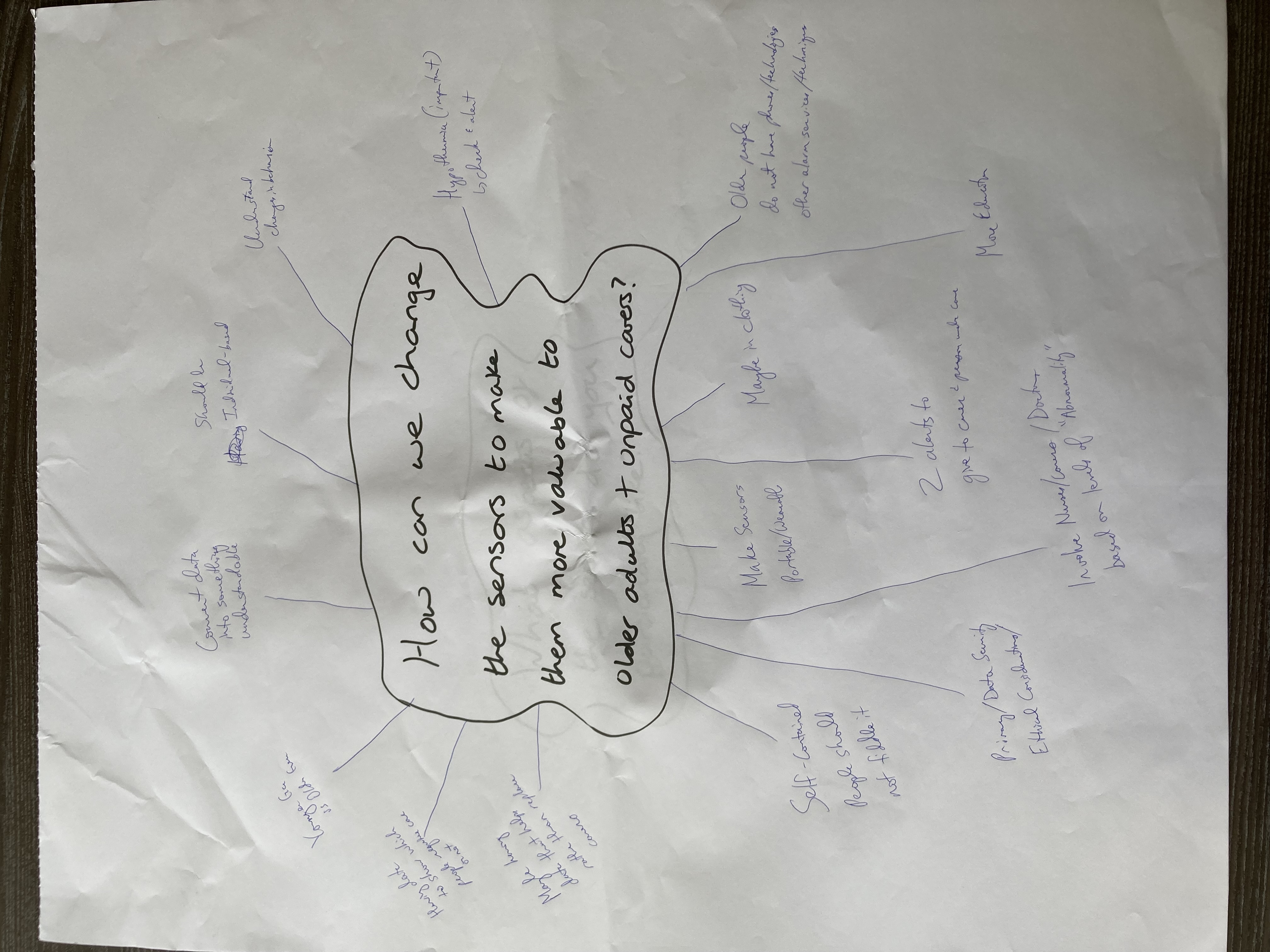

Supplement: Supplementary file 9 — Supplementary Material 9 [file 12877_2024_5674_MOESM9_ESM.zip › 12877_2024_5674_MOESM9/IMG_8178 - Copy.jpg]

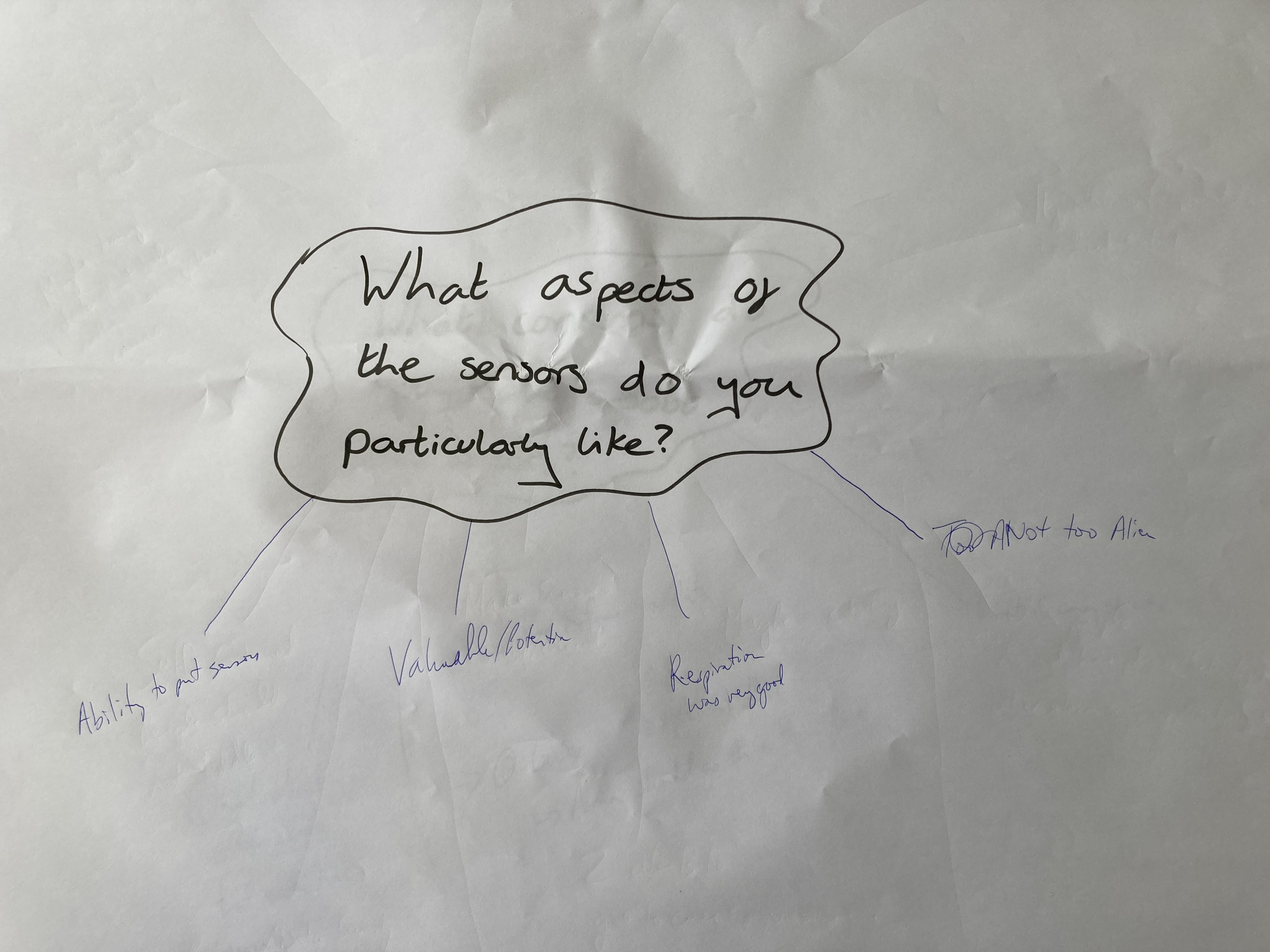

Supplement: Supplementary file 9 — Supplementary Material 9 [file 12877_2024_5674_MOESM9_ESM.zip › 12877_2024_5674_MOESM9/IMG_8179 - Copy.jpg]

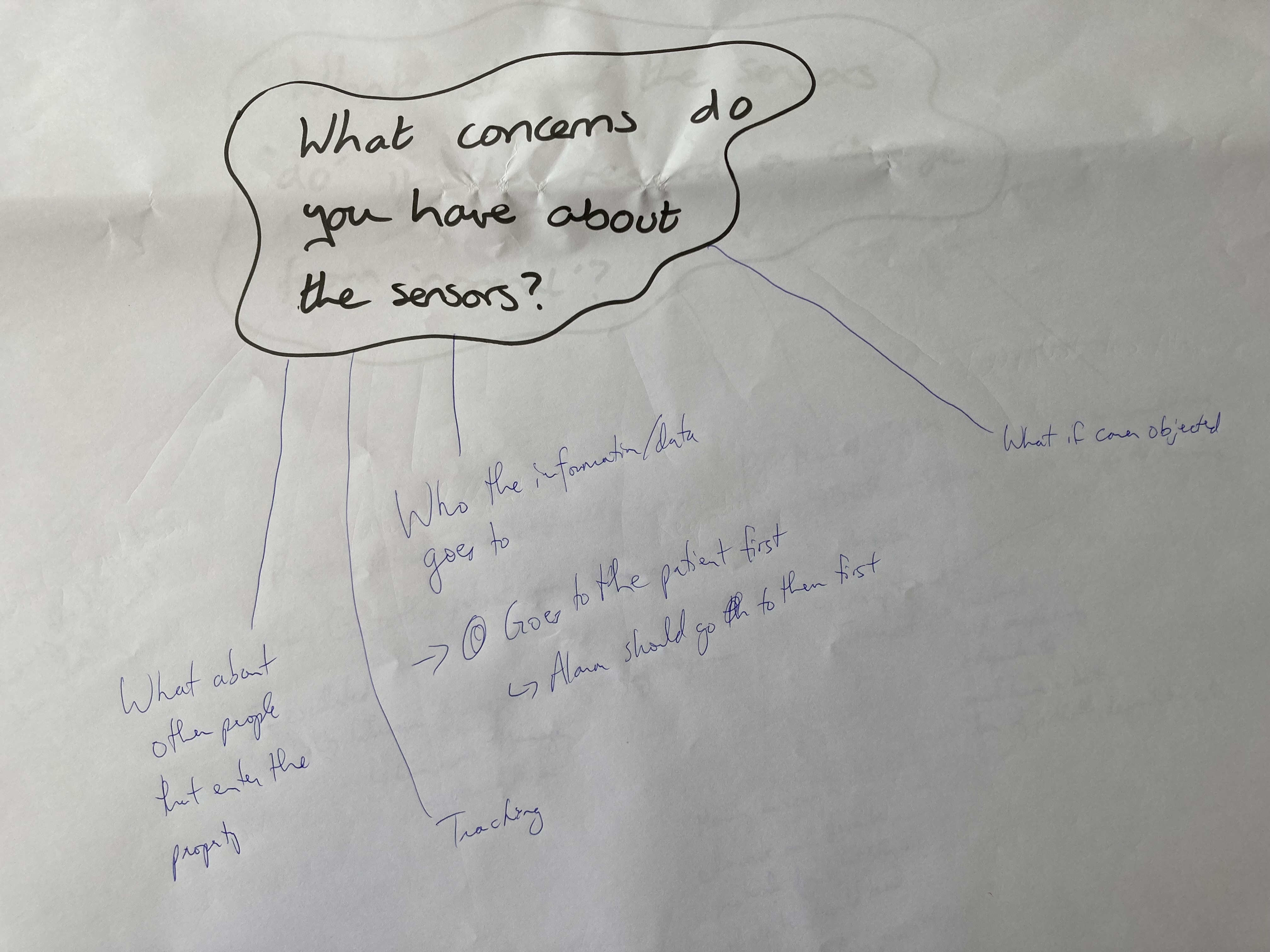

Supplement: Supplementary file 9 — Supplementary Material 9 [file 12877_2024_5674_MOESM9_ESM.zip › 12877_2024_5674_MOESM9/IMG_8180 - Copy.jpg]
